# A cross-sectional study on patient-centered outpatient service quality in the county-level integrated health organization of Yuhuan, Zhejiang province of China

**DOI:** 10.3389/fpubh.2026.1889257

**Published:** 2026-09-23

**Authors:** Qin Wang, Qihao Shen, Liming Shao, Qing Xu, Yijing Xie

**Affiliations:** 1 School of Public Health, Hangzhou Medical College, Hangzhou, Zhejiang, China; 2 School of Medical Humanities and Management (School of General Practice Competence Education), Hangzhou Medical College, Hangzhou, Zhejiang, China; 3 School of Population Medicine and Public Health, Chinese Academy of Medical Sciences and Peking Union Medical College, Beijing, China; 4 School of Education, Central China Normal University, Hangzhou, Zhejiang, China; 5 Institute of Health Policy, Hangzhou Medical College, Hangzhou, Zhejiang, China

**Keywords:** mediation effect, outpatient service quality, patient expectation and perception, patient intention to seek medical treatment, patient-centered, the county-level integrated health organization

## Abstract

**Purpose:**

This study aims to investigate the outpatient service quality of the Yuhuan CIHO and the interrelationships among patient expectations, perceived service quality, satisfaction, and behavioral intentions.

**Patients and methods:**

Data were analyzed using descriptive statistics, non-parametric tests, Wilcoxon signed-rank test, and multiple regression analysis to assess the status and influencing factors of outpatient service quality. Structural equation modeling (SEM) was applied to test the hypothesized relationships among expectations, perceptions, satisfaction, and behavioral intentions.

**Results:**

Patients’ socio-demographic characteristics and healthcare utilization patterns significantly influenced their expectations and perceptions of service quality. Overall, the service environment, facilities, and professional competence in the Yuhuan Health Community met or exceeded patient expectations. However, gaps between expectation and perception persisted in responsiveness and assurance at county-level hospitals, and in reliability at certain primary care institutions. SEM results revealed that while patient expectations did not directly influence satisfaction, they exerted a strong positive effect on perceived service quality (*β* = 0.953). Perceived service quality had a significant direct positive impact on patient satisfaction (*β* = 0.748). And patient satisfaction served as a key mediator.

**Conclusion:**

From a patient-centered perspective, health community development should prioritize reducing inter-institutional disparities in service delivery, actively manage patient expectations, and deliver personalized outpatient services. Furthermore, leveraging digital health platforms to strengthen vertical integration between county hospitals and primary care units is essential. Standardized protocols and proactive expectation management mechanisms should be established for critical dimensions such as responsiveness, assurance, and reliability to systematically enhance outpatient service quality.

## 1 Introduction

The assessment of healthcare service quality requires consideration of two fundamental dimensions: “technical quality” and “functional quality” (1). Gronroos pointed out that technical quality relates to the accuracy and effectiveness of diagnostic and treatment plans, while functional quality concerns the manner in which services are delivered, including staff attitudes, communication efficiency, and environmental comfort (2). These two dimensions together form the overall framework of healthcare service quality and are closely linked to patient experience. Patient experience refers to all the feelings and interactions patients undergo throughout their healthcare journey, from initial contact to follow-up care (3), encompassing evaluations of a healthcare institution’s clinical capabilities, as well as multiple aspects such as staff attitudes, doctor-patient communication, environmental comfort, process convenience, and emotional support (4). A study has shown that regularly collecting, thoroughly analyzing, and promptly responding to patient feedback and suggestions during the care process can significantly improve service workflows, enhance communication efficiency between doctors and patients, and effectively elevate both healthcare quality and patient satisfaction (5). High-quality patient experiences can significantly bolster patients’ trust in medical institutions, facilitate the transition of doctor-patient relationships towards a collaborative and partnership-oriented model, and consequently lay a solid groundwork for long-term health management (6). Before patients seek medical treatment, they anticipate experiencing timely, effective, and safe healthcare services throughout the medical process and receiving personalized healthcare services that respect their individual wishes (7). Patient Expectations refer to what individuals anticipate from medical services, and these expectations have an impact on their assessment of medical quality (8). It primarily encompasses various aspects, including the technical quality of healthcare services, service attitude, and the medical environment. Perception represents the patient’s immediate cognitive feedback on the service situation (9). Perception refers to the patient’s immediate cognitive feedback regarding the service situation (10). Studies from both China and other countries have shown that patient satisfaction is determined by the discrepancy between patient expectations and perceptions. Patient satisfaction increases significantly when perceived service quality exceeds expectations (11, 12). Patient satisfaction is a subjective evaluation by patients of the extent to which healthcare services meet their needs and expectations (13), which reflects the overall level of recognition by patients of the medical services provided by healthcare professionals. In outpatient settings, where contact time is brief and the service pace is rapid, even minor oversights in the medical visit process can be amplified. This underscores the need for healthcare institutions to carefully manage patient expectations and optimize the patient experience at critical touchpoints (14).

Patient satisfaction further affects patients’ behavioral intentions, including their willingness to revisit the same healthcare institution or recommend it to others (15). Patient behavioral intention refers to the tendency of patients to choose or utilize a specific type of medical service. With the growing availability of information channels, patients’ autonomy in selecting healthcare institutions has been steadily increasing (16). The higher the level of patient satisfaction, the more likely patients are to revisit the healthcare institution or recommend it to others.

The SERVQUAL model is the most widely used instrument for assessing patients’ perceived and expected service quality in healthcare settings. This study adopted the SERVQUAL outpatient service quality questionnaire refined by Chakravarty and other scholars (17), which comprises five first-level dimensions and 22 measurement items. These five dimensions—tangibility, reliability, responsiveness, assurance, and empathy-comprehensively capture patients’ multidimensional perceptions of outpatient services (18). This scale assesses service quality by measuring the gap between patients’ expectations and perceptions, transforming subjective experiences into measurable and actionable indicators, thereby enabling hospital managers to identify their strengths and areas for improvement (19). This model has been widely applied in evaluating healthcare services, but most studies have focused on single hospitals or specific clinical departments, with insufficient attention to patient experience within integrated health organizations covering county, township, and village levels. There is also limited research on patient experience assessments across different types of institutions within the same integrated system, such as county hospitals and primary healthcare centers.

Outpatients are the central focus of outpatient medical services, and the outpatient department serves as their first point of contact when seeking healthcare. The quality of outpatient services influences patients’ experiences across multiple dimensions—including medical technology, service processes, staff attitudes, and the clinical environment—and is closely linked to patients’ initial perceptions of the broader healthcare system as well as their subsequent behavioral intentions (20, 21). Improving outpatient service quality is essential for enhancing patients’ medical experiences, optimizing the utilization of medical resources, and fostering harmonious doctor–patient relationships (15, 22). Related studies have shown that primary care services lead to higher patient satisfaction than hospital outpatient services [Hammoudeh et al. (23)]. Other researcher [Guadie et al. (24)] focuses on evaluating service quality from the patient’s perspective, enhancing patient satisfaction through multiple dimensions such as patient experience and service processes. However, outpatient services currently face several challenges, including indifferent attitudes among some healthcare providers, sub-optimal service efficiency, ambiguous signage for registration and treatment procedures, inadequate patient privacy protection, and excessively high patient expectations, collectively revealing critical gaps in medical service delivery (25, 26).

The development of integrated healthcare models worldwide has provided an institutional framework for improving the quality of care from a patient-centered perspective. In the United States, the Patient-Centered Medical Home (PCMH) emphasizes team-based care led by primary care physicians, coordinated referrals, and a payment model combining fee-for-service with per-member-per-month management, covering enrolled patient populations. In contrast, Accountable Care Organizations (ACOs) consist of hospitals, physicians, and community service providers who collaborate under contract to take responsibility for the full continuum of care and total costs for specific insured populations (27, 28). To enhance the quality of primary healthcare services, China has also been gradually exploring approaches to improving patient experience and continuity of care within the framework of medical communities. The County Integrated Health Organization (CIHO) is a tightly integrated health community established under the coordination of county governments, led by county hospitals and jointly involving township health centers and village clinics. It operates with unified management of personnel, finances, and resources, and adopts a payment mechanism based on bundled prepayment of medical insurance funds, with surplus retained and excess costs shared. The service coverage spans the entire chain of prevention, treatment, rehabilitation, and health management (29). To date, research on CIHO has primarily focused on organizational structure and service models, with limited attention paid to patient experience. Understanding patients’ expectations and perceptions across different types of institutions within the integrated system can provide evidence for resource allocation strategies and help narrow the quality gap between county hospitals and primary healthcare centers.

In recent years, the reform of primary medical institutions in China has been progressively deepened, with increased emphasis placed on county-level public healthcare facilities. The establishment of medical communities has been actively promoted to integrate medical and health resources across county, township, and village levels. According to the “2024 Statistical Bulletin on the Development of China’s Health and Wellness Industry,” outpatient visits to primary and secondary hospitals and grassroots medical and health institutions reached 6.76 billion, accounting for 66.6% of the total (30). This represents a year-on-year increase of 350 million visits, reflecting growing public reliance on these services.

As the primary providers of grassroots medical services, county-level public hospitals play a pivotal role in determining the quality of outpatient care, which directly influences the health and well-being of the general population. Continuously improving the outpatient medical experience in county-level public hospitals to meet the growing public demand for higher-quality healthcare services has become a key objective of healthcare reform. As one of the first county-level health community alliances to implement reforms in Zhejiang Province, the Yuhuan People’s Hospital Health Community Alliance Group in Taizhou City (hereinafter referred to as “Yuhuan CIHO”) upholds a people-centered approach to health, adopts chronic disease management as its entry point, and is actively exploring an integrated three-in-one health service model encompassing “prevention, treatment, and rehabilitation (31).”

This study collected data on the gap between patients’ expectations and their actual experiences of medical services to evaluate the outpatient service quality of the Yuhuan CIHO. It analyzed the key factors influencing outpatient service quality and explored the moderating role of patient satisfaction, proposing targeted improvement measures to support the optimization of outpatient service processes and enhance overall service quality.

## 2 Research model and hypotheses development

The research model developed in this study primarily examines the relationships among patients’ expectations of outpatient service quality, their perceived actual service quality, patient satisfaction, and healthcare-seeking behavior. It further investigates how patient satisfaction mediates the influence of service quality perceptions on patients’ healthcare-seeking behavior (32). This study proposes the following hypotheses (Figure 1):

**Figure 1:**
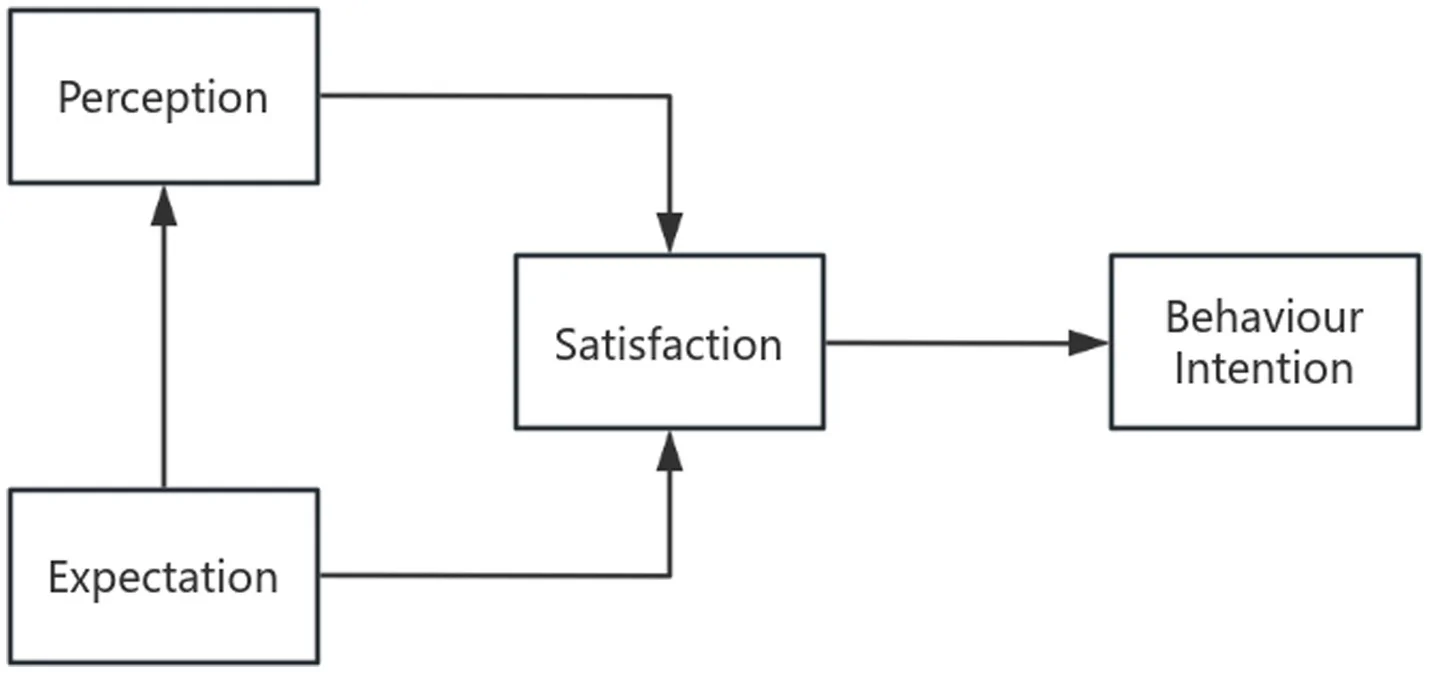
The relationship among expectations, perceptions, patient satisfaction, and patient willingness to seek medical care.

*H1*: There is a positive correlation between patients’ expectations of outpatient service quality and their perceived outpatient service quality.

Patients’ expectations regarding outpatient service quality encompass various aspects, including service facilities, the professional competence of medical staff, and response speed. The actual perceived service quality by patients is based on their real-life experiences during the healthcare process (33). According to the Service Quality Gap Model, customer expectations serve as the benchmark for service evaluation. Prior to receiving medical services, patients form expectations based on past experiences, word-of-mouth, and external information. During the service encounter, they compare their actual experience with these expectations, resulting in evaluations indicating whether the experience meets or exceeds expectations, or falls short. Total Quality Management theory emphasizes that understanding customer expectations is a prerequisite for delivering high-quality services. When patients perceive the quality of medical services to be higher than expected, they are more likely to express satisfaction. Based on this reasoning, the above hypothesis is proposed.

*H2*: There is a positive correlation between patients’ expectations and perceptions of outpatient service quality and patient satisfaction.

The Service Quality Gap Model suggests that the gap between expectations and perceptions influences patients’ overall service quality evaluations. Patient satisfaction reflects their comprehensive assessment of outpatient service quality. Research has shown that when there is a positive gap between initial expectations and actual perceptions—i.e., when perception exceeds expectations—patient satisfaction significantly increases (34, 35); conversely, a negative gap may lead to decreased satisfaction. Based on this, the above hypothesis is proposed.

*H3*: There is a positive correlation between patient satisfaction and patient healthcare-seeking behavior.

Total Quality Management theory posits that customer satisfaction is a key factor influencing quality management. A satisfying healthcare experience may encourage patients to continue seeking care at the same CIHO outpatient clinic and even recommend it to others; conversely, dissatisfaction may lead to patient attrition and switching to other healthcare providers. Empirical studies across various types of medical institutions have consistently found a positive association between satisfaction and healthcare-seeking behaviors (36, 37). Based on this, the above hypothesis is proposed.

*H4*: Patient satisfaction mediates the relationship between patient experience and healthcare-seeking behavior.

Building upon the previous three hypotheses (H1, H2, H3), this study further proposes that patient satisfaction plays a mediating role between patient experience and healthcare-seeking behavior. Expectation-Confirmation Theory (ECT) suggests that satisfaction arises from individuals comparing their pre-use expectations with their post-use performance perceptions (38). When perceived performance meets or exceeds expectations, individuals tend to feel satisfied; otherwise, dissatisfaction ensues. Patients’ perceptions of service quality during their visit influence their satisfaction or dissatisfaction, which in turn affects their willingness to continue using the same institution or recommending it to others. Agyapong et al. (39), in a study of 35 healthcare institutions in Ghana, confirmed that satisfaction mediates the relationship between perceived service quality and behavioral intention. Chocknakawaro and Pusaksrikit, (40), in a study of public university hospitals in Thailand, found that patient satisfaction mediated the relationships between five dimensions of service quality and patient loyalty. This study aims to test these relationships within the context of CIHO, analyzing the mediating role of satisfaction under the CIHO framework. Based on this, the above hypothesis is proposed.

Next, this study will conduct empirical analyses to test the validity of these hypotheses.

## 3 Methods and materials

### 3.1 Research design and setting

From May to August 2025, a cross-sectional study was conducted in the Yuhuan CIHO, Zhejiang Province, China. The Yuhuan CIHO is an integrated healthcare system led by Yuhuan People’s Hospital—a tertiary grade B general hospital—that oversees five township health centers located in Kanmen, Damaqu, Haicheng, Yucheng, and Lupu. This structure constitutes a three-tier urban–rural integrated health service network characterized by a clear hierarchical organization and functionally complementary units, designed to deliver comprehensive, continuous, and efficient medical services to local residents. This study employed a cluster sampling method, selecting all six member institutions of the Yuhuan City CIHO as survey sites, and choosing three outpatient departments—internal medicine, surgery, and general practice—as the target departments. These departments have relatively high daily patient volumes and broad coverage of patient populations, making them highly representative across the member institutions and capable of comprehensively reflecting the healthcare experiences and service quality evaluations of patients from different types of outpatient settings.

### 3.2 Participants and respondents

A total of 1,033 questionnaires were collected. The inclusion criteria for this study were patients who had sought outpatient care within the Yuhuan CIHO in the past year and who were capable of independently and accurately reporting their clinical visit experiences. This research excludes individuals who were unable to provide objective and rational evaluations due to severe physical illness or cognitive/mental impairments that could affect judgment. Prior to data collection, trained investigators provided a detailed explanation of the study’s purpose and procedures to all participants. The study was approved by the medical ethics committee of Hangzhou Medical College [LL2025-002], and informed consent was obtained from all participants.

### 3.3 Check list developments

This study utilized a modified version of the SERVQUAL instrument to assess outpatient service quality (41). Parasuraman et al. (42) explicitly pointed out that the dimensions of SERVQUAL can be adapted according to specific service contexts. Medical services differ from general commercial services, and patients also place high importance on safety assurance during emergencies. CIHO integrates county-level hospitals with primary healthcare institutions, covering a broad service area and handling large patient volumes. As such, outpatient safety management and order maintenance have become critical factors influencing patients’ medical experience; therefore, this questionnaire has added an item assessing emergency response capability (18).

The questionnaire comprises two sections. Section one collects patient socio-demographic information, including gender, age, education level, household registration status, and monthly income. Section two contains 23 items organized across five established dimensions of service quality (Table 1): (1) Tangibility—evaluating the physical appearance of facilities, equipment, and personnel, as well as the alignment between visible elements and service expectations; (2) Reliability—measuring the ability of the healthcare system to consistently deliver promised services accurately and on time; (3) Responsiveness—assessing the promptness and willingness of staff to assist patients upon request; (4) Assurance—reflecting the competence, professionalism, and courtesy of providers that foster patient trust and confidence; and (5) Empathy—capturing the extent to which personalized and compassionate care is provided from the patient’s perspective.

**Table 1:** Evaluation index of CIHO’s outpatient service quality.

Project	Dimension	No	Item
Expectation	Tangibles	T1_E	If service facilities (e.g., self-service registration machines) are adequate and well maintained.
		T2_E	If medical staff are professionally dressed.
		T3_E	If the signage is clear and easy to locate.
		T4_E	If health education materials are available at the waiting area for patients.
		T5_E	If the waiting and consultation areas are clean, well maintained, and properly ventilated.
	Reliability	R1_E	If electronic medical records are clearly documented and easyly accessible.
		R2_E	If doctors provide accurate diagnoses.
		R3_E	If patients’ communication with medical staff are adequate, smooth and effective
		R4_E	If all items and charges on medical bills are transparent and clearly explained.
		R5_E	If patients receive continuous and coordinated medical care.
	Responsiveness	Rs1_E	If rotating medical staff are available at the information desk to provide timely assistance.
		Rs2_E	If a clear complaint-handling system is in place, and complaints are addressed promptly.
		Rs3_E	If outpatient services are organized and provided in a timely manner.
		Rs4_E	If examination results and medication pickup reminders are provided promptly.
		Rs5_E	If the outpatient department has effective emergency response capabilities (e.g., security management and police patrols).
	Assurance	A1_E	If medical staff possess professional knowledge and proficient clinical skills.
		A2_E	If examination results are recognized within the hospital, reducing unnecessary tests.
		A3_E	If doctors carefully and patiently inquire about patients’ conditions and clearly explain treatment plans, medication use, and dietary or rehabilitation advice.
		A4_E	If medical staff and other hospital personnel demonstrate a positive and respectful attitude.
	Empathy	E1_E	If doctors provide personalized services tailored to patients’ needs.
		E2_E	If patients’ privacy is respected and protected.
		E3_E	If patients’ right to informed consent is respected.
		E4_E	If doctors prioritize patients’ feelings and best interests.
Perception	Tangibles	T1_P	If service facilities (e.g., self-service registration machines) are adequate and well maintained.
		T2_P	If medical staff are professionally dressed.
		T3_P	If the signage is clear and easy to locate.
		T4_P	If health education materials are available at the waiting area for patients.
		T5_P	If the waiting and consultation areas are clean, well maintained, and properly ventilated.
	Reliability	R1_P	If electronic medical records are clearly documented and easyly accessible.
		R2_P	If doctors provide accurate diagnoses.
		R3_P	If patients’ communication with medical staff are adequate, smooth and effective
		R4_P	If all items and charges on medical bills are transparent and clearly explained.
		R5_P	If patients receive continuous and coordinated medical care.
	Responsiveness	Rs1_P	If rotating medical staff are available at the information desk to provide timely assistance.
		Rs2_P	If a clear complaint-handling system is in place, and complaints are addressed promptly.
		Rs3_P	If outpatient services are organized and provided in a timely manner.
		Rs4_P	If examination results and medication pickup reminders are provided promptly.
		Rs5_P	If the outpatient department has effective emergency response capabilities (e.g., security management and police patrols).
	Assurance	A1_P	If medical staff possess professional knowledge and proficient clinical skills.
		A2_P	If examination results are recognized within the hospital, reducing unnecessary tests.
		A3_P	If doctors carefully and patiently inquire about patients’ conditions and clearly explain treatment plans, medication use, and dietary or rehabilitation advice.
		A4_P	If medical staff and other hospital personnel demonstrate a positive and respectful attitude.
	Empathy	E1_P	If doctors provide personalized services tailored to patients’ needs.
		E2_P	If patients’ privacy is respected and protected.
		E3_P	If patients’ right to informed consent is respected.
		E4_P	If doctors prioritize patients’ feelings and best interests.

Reliability analysis demonstrated high internal consistency for the measurement scales. Cronbach’s α coefficients were 0.981 for the expectation scale and 0.980 for the perception scale, indicating excellent overall reliability. At the dimension level, the expectation scale achieved *α* values of 0.930 (tangibility), 0.906 (reliability), 0.925 (responsiveness), 0.920 (assurance), and 0.859 (empathy). Corresponding values for the perception scale were 0.916 (tangibility), 0.922 (reliability), 0.929 (responsiveness), 0.908 (assurance), and 0.829 (empathy). While the empathy subscale in the perception scale exhibited a slightly lower coefficient, it remains within acceptable limits (>0.80); all other dimensions exceeded the recommended threshold of 0.85, confirming strong reliability at both the composite and domain levels (see Supplementary Tables 1–5).

### 3.4 Data collection and analysis

A total of 1,050 outpatients were enrolled in this study, and 1,033 valid questionnaires were collected, yielding an effective response rate of 98.38%. The distribution of respondents across healthcare facilities was as follows: 522 from Yuhuan People’s Hospital, 79 from Damaiyu Township Health Center (THC), 132 from Haishan THC, 120 from Kanmen THC, 92 from Lupu THC, and 88 from Yucheng THC. Data were collected through a combination of face-to-face interviews and online surveys. All survey data were independently double-entered by five trained research staff using EpiData software, with cross-validation performed to ensure data accuracy and completeness.

### 3.5 Descriptive and statistic analysis

This study utilized R software (version 3.5.0) (43) to analyze patient socio-demographic characteristics and outpatient service quality. To examine differences in patients’ expectations and perceptions across the five dimensions of service quality by age group, sex, education level, occupation type, monthly income, household registration, insurance type, and frequency of healthcare visits, the Kruskal-Wallis H test was employed. To assess whether the gap between patients’ expected and perceived service quality scores was statistically significant, the Wilcoxon signed-rank test was employed. This non-parametric test was chosen for two reasons: (1) expectation and perception scores were paired—each patient rated the same 23 service items twice, once for expectations and once for actual perceptions; and (2) the Likert-scale response data (1–5) did not satisfy the normality assumption required for parametric paired *t*-tests. Results are presented as medians with interquartile ranges (Q1, Q3), and the Wilcoxon *W* statistic is reported. This study employs stepwise multiple linear regression, using perceived service quality across five dimensions as the dependent variable and demographic information along with expectations across five dimensions as independent variables, to analyze factors influencing patients’ perceived gaps in service quality. Given a large sample size, we aim to identify the most closely related predictors among multiple potential variables affecting perceived service quality.

### 3.6 SERVQUAL model

This study employed the SERVQUAL model to conceptualize service quality across five core dimensions. Tangibility refers to the visual appeal of physical facilities, equipment, personnel, and communication materials (44), encompassing service infrastructure, healthcare staff attire, wayfinding signage, and the environment of waiting and consultation areas. Reliability denotes the ability to consistently and accurately deliver promised services (45). Responsiveness reflects the willingness and promptness of service providers to assist patients. Assurance encompasses the knowledge, competence, courtesy, and credibility of staff that inspire patient trust and confidence. Empathy refers to individualized care and the extent to which providers understand and address patients’ unique needs. For each dimension, the mean score of its constituent items was computed to represent overall performance. Each item was rated twice—once to capture patient expectations and once to assess patient perceptions. The perception–expectation gap (P − E) for each dimension was calculated by subtracting the expectation score from the corresponding perception score (46), serving as an indicator of outpatient service quality (47).

### 3.7 Mediation analysis

To further investigate the interrelationships among patient expectations and perceptions, patient satisfaction, and willingness to seek medical care, this study applied structural equation modeling (SEM) using AMOS 21.0 for hypothesis testing. Confirmatory factor analysis (CFA) was performed to assess the validity and reliability of the measurement model, followed by evaluation of the initial theoretical model. Path coefficients were estimated using maximum likelihood estimation, and hypotheses were tested based on standardized regression weights. Model fit was evaluated through multiple goodness-of-fit indices (e.g., CFI, TLI, RMSEA, SRMR), and the model was iteratively refined by incorporating theoretically justified modifications. The final modified model was re-evaluated, and the significance of path coefficients was confirmed. Additionally, the mediating effect of patient satisfaction was examined using bootstrapping procedures to test indirect effects.

## 4 Results

### 4.1 Sociodemographic characteristics of respondents

The demographic analysis of the patients surveyed revealed a fairly equal distribution of gender among the surveyed patients and a mean age of 41.01 years. The 20–59 years age group had the most patients (840 patients, 81.3%) and the high school or more educational attainment (788 patients, 76.58%). Most people work in service industry jobs, corporate jobs and professional tech jobs. Of those, 622 (60.44%) had a monthly income of less than 5,000 yuan, while 424 (41.20%) were covered under urban medical insurance (Table 2).

**Table 2:** Demographic characteristics of the participants.

Project	Dimension	Count	Column *N* %
Age	<40	504	49.0%
	40–59	336	32.7%
	>60	189	18.4%
Sex	Male	490	47.4%
	Female	543	52.6%
Education degrees	Primary school or below	245	23.7%
	Middle school	466	45.1%
	College or above	322	31.2%
Job	Famer	102	9.87%
	Famer	102	9.9%
	P&T Equip. Ops. & Assoc. Staff	105	10.2%
	Bus. & Serv. Staff	229	22.2%
	Admin. Staff	32	3.1%
	Ent. Staff	110	10.6%
	Prof. Tech. Staff	89	8.6%
	Soldier	10	1.0%
	Student	172	16.7%
	Retire	104	10.1%
	Unemp.	80	7.7%
Income	<2,000	245	23.7%
	2,000–5,000	377	36.5%
	5,000–10,000	356	34.5%
	>10,000	55	5.3%
Medical insurance	medical insurance for urban workers	424	41.0%
	Urban workers’ medical insurance	234	22.7%
	New Rural Cooperative Medical Scheme	200	19.4%
	commercial insurance	91	8.8%
	Student medical insurance	84	8.1%
Household registration	Rural	562	54.4%
	Urban	471	45.6%
Hospital	Yuhuan People’s Hospital (Y1)	522	50.53%
	Damaiyu THCs (Y2)	79	7.65%
	Haishan THCs (Y3)	132	12.78%
	Kanmen THCs (Y4)	120	11.62%
	Lupu THCs.(Y5)	92	8.91%
	Yucheng THCs (Y6)	88	8.52%
Outpatient department	Internal	195	18.9%
	Surgery	206	19.9%
	General	632	61.2%
Number of visits	1–5	560	54.2%
	6–10	210	20.3%
	11–15	175	16.9%
	16+	88	8.5%

P&T Equip. Ops. & Assoc. Staff, Production; Transportation & Equipment Operators; and Associate Staff; Bus. & Serv. Staff, Business & Service Staff; Admin. Staff, Administrative Staff; Ent. Staff, Entertainment Staff; Prof. Tech. Staff, Professional & Technical Staff; URBMI, Urban and Rural Residents Basic Medical Insurance; UWM, Urban Employee Basic Medical Insurance; NCMS, New Rural Cooperative Medical System; CI, Commercial Insurance; SMI, Student Medical Insurance.

Gaps between expectation and perception of service quality according to patients’ demographic characteristics.

As demonstrated in Supplementary Table 6, there are significant age-related differences regarding the dimension of empathy (*p* < 0.001) among the five perceived dimensions. There were statistical gender differences in the dimensions of tangibility (*W* = 145,515, *p* = 0.008), reliability (*W* = 146146.5, *p* = 0.005), and assurance (*W* = 144451.5, *p* = 0.015) of both expectations and perceptions. Overall, male patients keep scoring significantly higher than female patients in quite a number of dimensions. There were significant differences (*p* < 0.001) in patients’ expectations and perceptions in all five dimensions across education level, occupation type, monthly income and medical insurance type. The statistical difference between urban and rural settings was seen to be significant only on the dimension of expected responsiveness (*W* = 123,015, *p* = 0.047). Significant differences in five dimensions of expectations and perceptions were present between patients from different hospitals and departments, as well as patients with various numbers of medical visits (*p* < 0.001).

Radar charts were plotted (Figure 2) to provide a visual comparison of the scores for six hospitals with respect to patients’ expectations and perceptions across different dimensions. This enables us to analyze the mean difference between expectations and perceptions. Results of data analyses for each hospital are indicated in Table 3. Hospital 1 scored lower than expected in both responsiveness (*W* = 20725.500, *p* = 0.002) and assurance (*W* = 16725.000, *p* = 0.034). As the leading county-level hospital within the CIHO system, Hospital 1 handles complex medical services and a high volume of outpatient visits. The gaps in responsiveness and assurance suggest that patients still perceive room for improvement regarding the efficiency of service processes, adequacy of communication, and confidence in treatment plans. Hospital 2 scored lower than expected in reliability (*W* = 1084.500, *p* = 0.040), indicating that as a primary healthcare institution, it falls short of patient expectations in terms of standardized service procedures and predictable clinical practices, which may reflect shortcomings in information technology development, standardization of care, and staff training. Hospital 3 scored higher than expected in assurance (*W* = 411.000, *p* = 0.007), suggesting that patients experienced greater-than-expected levels of effective communication, respectful attitudes, and trust in medical staff. Hospital 4 scored higher than expected in tangibility (*W* = 726.500, *p* = 0.006), indicating that patients are relatively satisfied with the physical facilities and overall environment at this primary healthcare institution.

**Figure 2:**
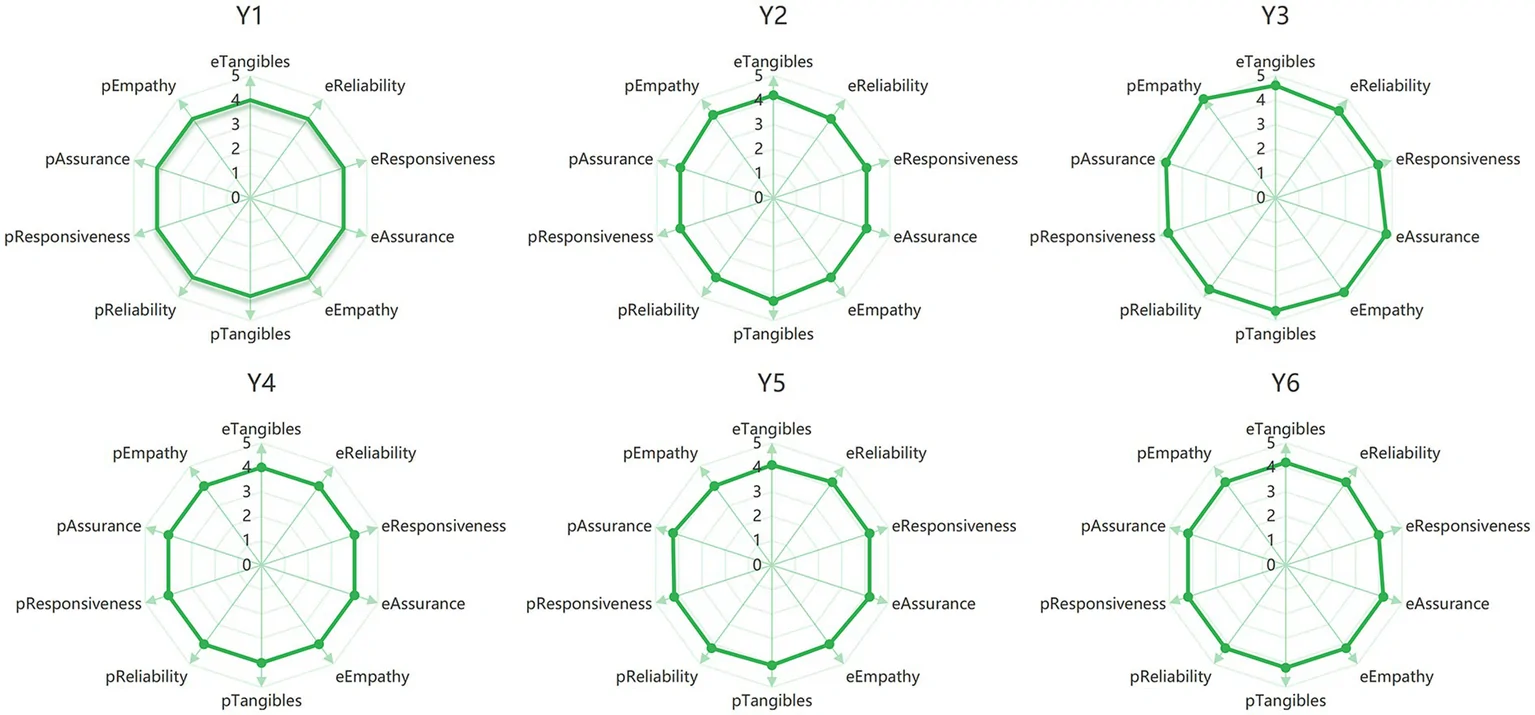
Radar chart of hospital-level expectation–perception gaps. Y1: Yuhuan People’s Hospital, Y2: Damaiyu THCs, Y3: Haishan THCs, Y4: Kanmen THCs, Y5: Lupu THCs, Y6: Yucheng THCs.

**Table 3:** Wilcoxon signed-rank test of patient expectation and perception of patient expectation and perception.

Hospital	Expectation Median (Q1, Q3)	Perception Median (Q1, Q3)	*W*	*p*
Responsiveness				
Y1 (*N* = 522)	4 (3.4, 5)	4 (3.4, 5)	20725.500	0.002^**^
Assurance				
Y1 (*N* = 522)	4 (3.5, 5)	4 (3.25, 5)	16725.000	0.034^*^
Y3 *(N* = 132)	4.75 (4, 5)	4.75 (4.25, 5)	411.000	0.007^**^
Reliability				
Y2 (*N* = 79)	4 (4, 4.6)	4 (3.6, 4.6)	1084.500	0.040^*^
Tangibles				
Y4 (*N* = 120)	4 (3.75, 4.4)	4 (4, 4.6)	726.500	0.006^**^

Y1, Yuhuan People’s Hospital; Y2, Damaiyu THCs; Y3, Haishan THCs; Y4, Kanmen THCs; *p < 0.05, **p < 0.01, ***p < 0.001.

### 4.2 Wilcoxon signed-rank test of expectations and perceptions

The objective of this study is to analyze the dissimilarities between the latent and actual ability levels with respect to various outpatient visiting dimensions at the different levels of healthcare institutions of the Yuhuan Healthcare Group. The findings in Table 3 demonstrate that Hospital 1 patients’ perceived scores in the responsiveness (*W* = 20725.500, *p* = 0.002) and assurance (*W* = 16725.000, *p* = 0.034) dimensions were significantly lower than their expected scores. In Hospital 2, patients’ perceived score was significantly less than their expected score in reliability (*W* = 1084.500, *p* = 0.040). At Hospital 3, the perceived scores of the patients were significantly higher than their expected scores in the assurance dimension (*W* = 411.000, *p* = 0.007). The patients of Hospital 4 obtained significantly higher perceived scores compared to the expected scores (*W* = 726.500, *p* = 0.006) under the tangibility dimension (see Supplementary Table 7).

### 4.3 Predictors of patient perception in outpatient service quality

The findings revealed that expected tangibility had a significant and positive prediction of perceived tangibility (*β* = 0.83, 95% CI: 0.79–0.86, *p* < 0.001, Supplementary Table 8). The perceived tangibility score of NCMS participants was significantly lower than that of commercial insurance participants (*β* = −0.13, 95% CI: −0.20 to −0.05, *p* = 0.001) among the types of health insurance. It was found that expected reliability could significantly and positively predict perceived reliability (*β* = 0.79, 95% CI: 0.76–0.83, *p* < 0.001). Patients whose education level is limited to primary school or less had a higher perceived reliability score than those whose education level is college or more (*β* = 0.14, 95% CI: 0.05–0.22, *p* = 0.001). Expectation responsiveness had a significantly positive impact on perceived responsiveness at *β* = 0.85. In an analysis based on age category, patients aged 60 years and above showed higher perception responsiveness scores as compared to patients aged 20–39 years (*β* = 0.15, 95% CI: 0.06–0.24, *p* < 0.001). Regarding visit frequency, patients with 16 or more visits (*β* = −0.08, 95% CI: −0.16 to −0.00, *p* = 0.046) and those with 6–10 visits (*β* = −0.17, 95% CI: −0.28 to −0.07, *p* = 0.001) had significantly lower perceived responsiveness scores than those with 1–5 visits. Expectation assurance was found to be a significant positive predictor of perceived assurance (*β* = 0.75, 95% CI: 0.71–0.78, *p* < 0.001). Among hospital types, hospital 3 had an assurance score that was significantly different when compared to hospital 1 (*β* = 0.16, 95% CI: 0.06–0.27, *p* = 0.002). Expectation empathy was proven to be a strong positive predictor of perceived empathy (*β* = 0.74, 95% CI: 0.70–0.78, *p* < 0.001). Regarding department types, surgical patients were appreciated empathy scores significantly higher apparently than general practice patients (*β* = 0.11, 95% CI: 0.02–0.19, *p* = 0.014) department types. Regarding visit frequency, patients with 6–10 visits had significantly lower perceived empathy score compared to patients that had 1–5 visits (*β* = −0.19, 95% CI: −0·31 to −0·07, *p* = 0.001) (see Supplementary Table 8).

### 4.4 Mediation analysis

#### 4.4.1 Reliability and validity analysis

This research evaluated the internal consistency reliability, indicator reliability, convergent validity, and discriminant validity of the structural equation model (SEM). The indices of the model were sound with standardized factor loadings ranging from 0.817 (Empathy3_P), to 0.886 (Responsiveness1_P). All values surpassed the established threshold value of 0.70 set as an assessment criterion, indicating a high degree of reliability of the selected indicators. The composite reliability of each dimension varied from 0.866 to 0.901. The findings (Table 4) show that the Cronbach’s α coefficient for every latent variable exceeds 0.8. This signifies that the employed method or model has a high level of reliability and provides sufficient evidence for model construction.

**Table 4:** Reliability analysis of measurement variables (for details, see the Supplementary Table 9).

Project	Dimension	Cronbach’s alpha
Expectation	Tangibles	0.889
	Reliability	0.898
	Responsiveness	0.892
	Assurance	0.890
	Empathy	0.873
Perception	Tangibles	0.901
	Reliability	0.899
	Responsiveness	0.900
	Assurance	0.882
	Empathy	0.866

The study analyzes composite reliability (CR) using SEM. A CR value above 0.60–0.75 is considered acceptable. In this study, all the dimensions registered CR values from 0.8665 (Empathy_P) to 0.9015 (Tangibles_P), which are significantly higher than the threshold. Hence, it further confirms the model’s reliability. The average variance extracted AVE is an important measure of convergent validity. All values for AVE presented were in excess of 0.50, meaning all constructs possessed good convergent validity (Table 5).

**Table 5:** Validity (for details, see the Supplementary Table 10).

Project	Dimension	CR	AVE
Expectation	Tangibles	0.8904	0.67
	Reliability	0.8734	0.6331
	Responsiveness	0.8883	0.6143
	Assurance	0.898	0.6378
	Empathy	0.8924	0.624
Perception	Tangibles	0.8813	0.6501
	Reliability	0.8665	0.6187
	Responsiveness	0.9015	0.6469
	Assurance	0.8996	0.6419
	Empathy	0.9001	0.6432

#### 4.4.2 Hypothesis testing

Confirmatory measurement factor analysis was used in this study to evaluate the associations among indicators and the linear relationships between each path. The results of the study indicated that there are statistically significant relationships among the variables in the proposed model, as shown in Table 6. The results of the CFA indicated *χ*^2^ = 462.041, *χ*^2^/d*f* = 7.452, *p* < 0.001, TLI = 0.97, CFI = 0.976, GFI = 0.937, AGFI = 0.908, RMSEA = 0.079 (Figure 3).

**Table 6:** Path analysis for the initial SEM.

Initial SEM	B	*β*	S.E.	C.R.	*p*
Perception<---Expectation	0.971	0.955	0.021	46.046	***
Patients’ Satisfaction<---Perception	0.908	0.741	0.114	7.94	***
Patients’ Satisfaction<---Expectation	0.007	0.006	0.116	0.065	0.948
Behavioral Intention<---Patients’ Satisfaction	0.051	0.357	0.006	8.041	***

*p < 0.05, **p < 0.01, **p < 0.001.

**Figure 3:**
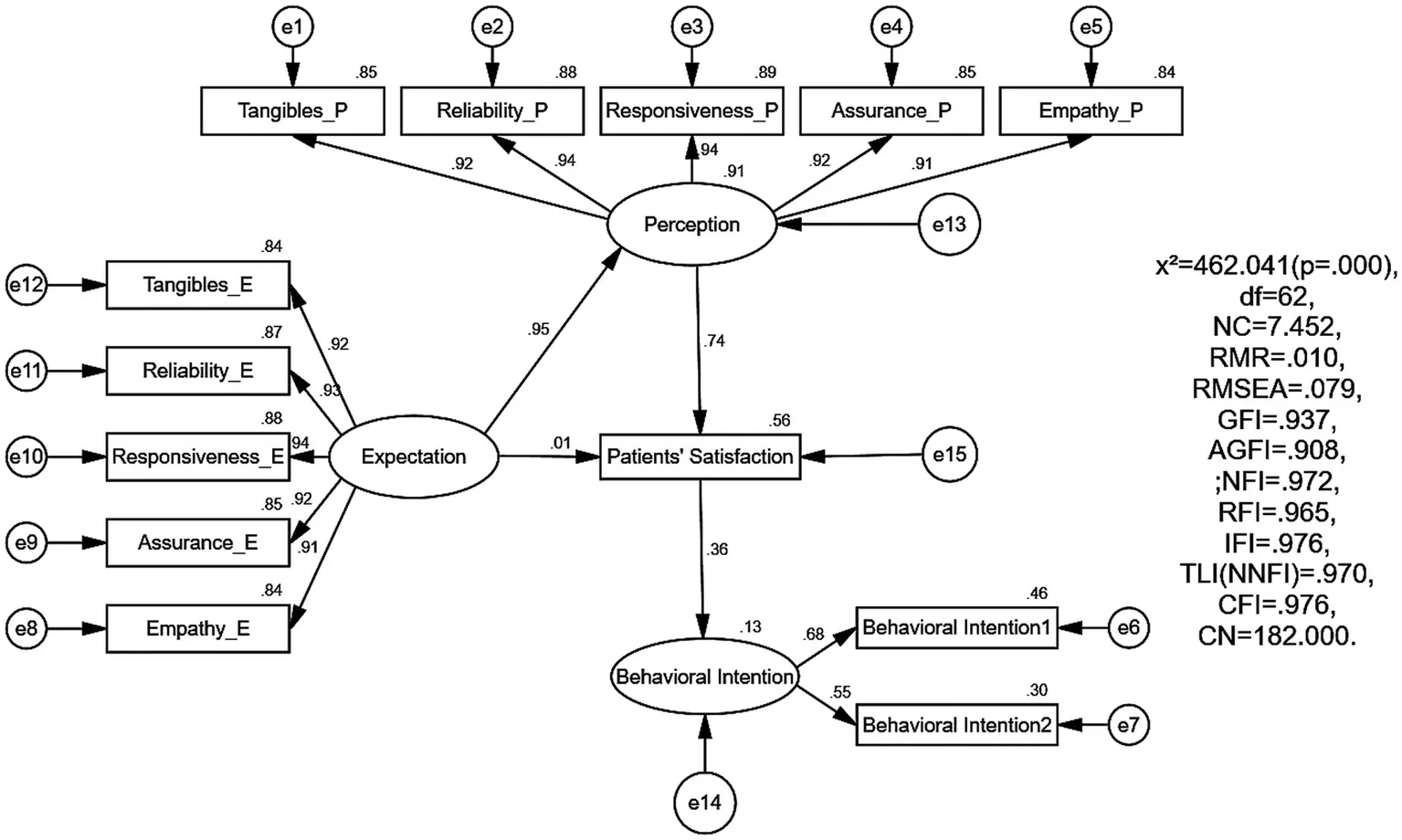
Initial structural equation model (SEM) of patient expectation, perception, satisfaction, and behavioral intention.

In the preliminary model, the path between patient expectations and patient satisfaction was not significant, while a direct relationship might exist between patient expectations and behavioral intentions. Based on modification indices (MI) and theoretical rationale, the relationship between patient expectations and patient satisfaction in the original model was revised in this study, and a path related to patient expectations and behavioral intentions was also added. In addition, confirmatory factor analysis (CFA) yielded the following results: *χ*^2^ = 368.41, *χ*^2^/d*f* = 6.244, *p* < 0.001, TLI = 0.975, CFI = 0.981, GFI = 0.949, AGFI = 0.922, RMSEA = 0.071, SRMR = 0.0154. While the Chi-square test remained statistically significant—reflecting the large sample size (*N* = 1,033) rather than substantive model misspecification—all other fit indices met or exceeded the recommended thresholds, indicating good model fit (Figure 4). Furthermore, patient satisfaction serves as a mediating variable between healthcare service quality and patient behavioral intentions (see Supplementary Tables 9,10).

**Figure 4:**
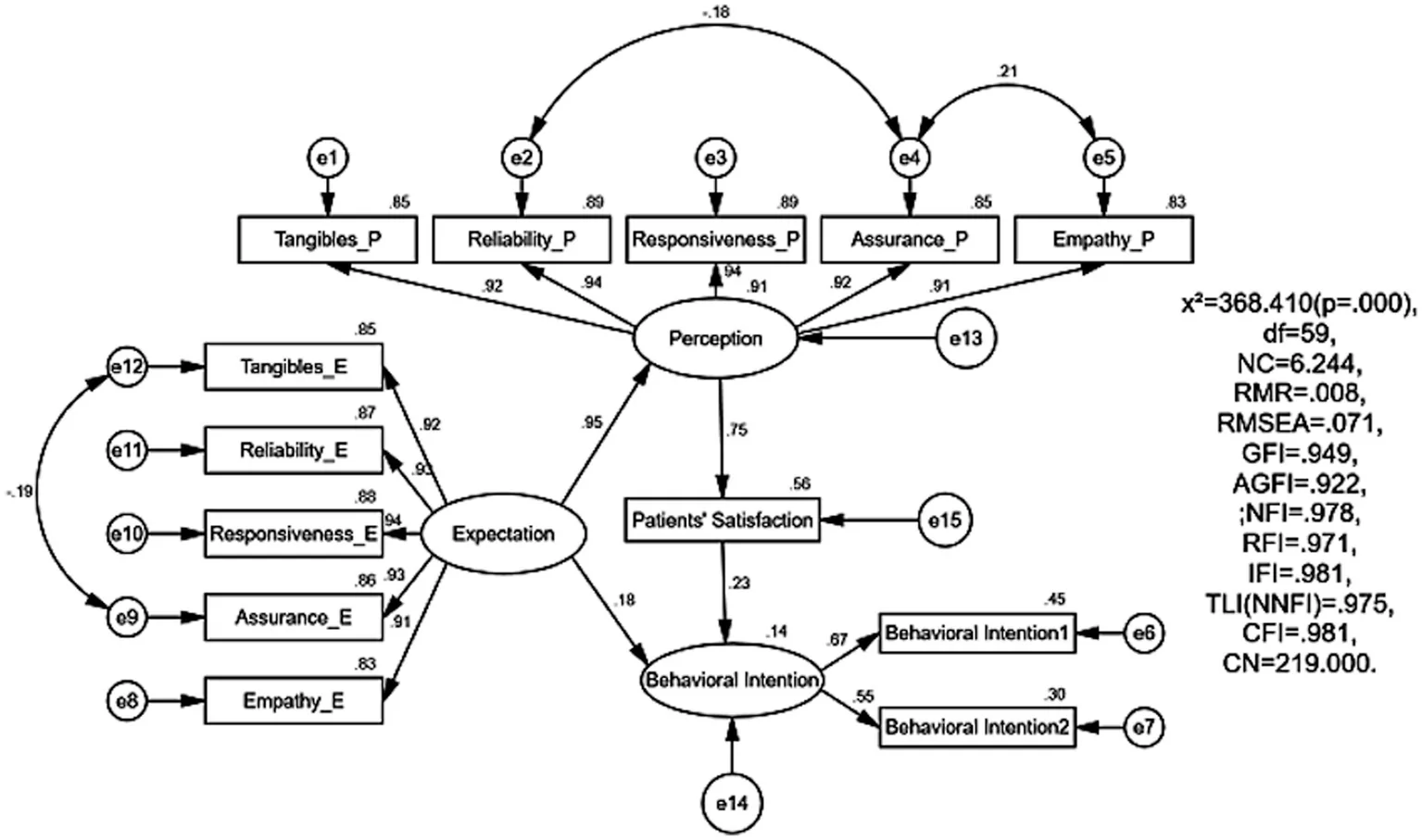
Revised structural equation model (SEM) of patient expectation, perception, satisfaction and behavioral intention.

In the structural equation model, this study tested the following hypotheses (see Table 7):

**Table 7:** Path analysis of the revised SEM.

Revised SEM	B	*β*	S. E.	C. R.	*p*
Perception<---Expectation	0.971	0.953	0.021	45.699	***
Patients’ Satisfaction<---Perception	0.916	0.748	0.028	32.394	***
Behavioral Intention<---Patients’ Satisfaction	0.032	0.229	0.008	3.89	***
Behavioral Intention<---Expectation	0.032	0.181	0.01	3.059	**
Behavioral Intention<---Perception	0.120	0.375	0.061	1.968	0.049*

*p < 0.05, **p < 0.01, **p < 0.001, S.E., standard error; C.R., critical ratio.

*H1*: There is a positive correlation between patients’ expectations of outpatient service quality and their perceived outpatient service quality.

The results show that the path coefficient from expectation to perception is *B* = 0.971, *β* = 0.953, with *p* < 0.01, exceeding the threshold for a large effect size (0.50) (Table 7). This indicates that the higher patients’ expectations regarding outpatient service quality, the higher their actual perceived ratings during healthcare encounters, supporting H1. The effect size also suggests that when patients’ perceived service quality increases by one standard deviation, their satisfaction scores are expected to increase by approximately 0.75 standard deviations. Focusing resources on areas where patient perception is weakest—such as responsiveness and assurance—may be more effective than comprehensive improvements across all aspects of outpatient services.

*H2*: Patients’ expectations and perceptions of outpatient service quality are significantly positively correlated with patient satisfaction.

The test results indicate that the path coefficient from expectation to satisfaction is 0.007, with *p* = 0.948, which is not significant at the 0.05 level; thus, there is no significant positive relationship between patients’ expectations of service quality and satisfaction. However, the path coefficient from perception to satisfaction is 0.916, with *p* < 0.01, indicating a statistically significant positive relationship between perceived service quality and satisfaction (Table 7). This effect size is also considered large, meaning that for every one standard deviation increase in perceived service quality, satisfaction is expected to rise by approximately 0.75 standard deviations (*β* = 0.748) (Table 7). This finding suggests that focusing improvement efforts on the dimensions where patient perception is weakest—such as responsiveness and assurance—could be more effective than broad-based service enhancements. Thus, the portion of H2 concerning the relationship between perception and satisfaction is supported.

*H3*: Patient satisfaction is significantly positively correlated with patient healthcare behavior.

The results show that the path coefficient from satisfaction to behavioral intention is 0.229 (*p* < 0.001), reaching statistical significance, with a small to moderate effect size. In the context of medical services, which are subject to multiple institutional constraints such as insurance coverage, referral regulations, and geographic accessibility, satisfaction still exerts a stable incremental predictive effect on behavioral intentions. This implies that patients’ emotional experiences can, to some extent, overcome institutional barriers and influence their behavioral choices. Therefore, managers may gain marginal benefits at the behavioral level by improving patient satisfaction. H3 is supported.

*H4*: Patient satisfaction mediates the relationship between patient experience and healthcare behavior.

To test the mediating effect of patient satisfaction, the results are presented in Table 7. The indirect effect of perception on behavioral intention through satisfaction was calculated as the product of the path coefficients: Perception → Satisfaction (*β* = 0.748) and Satisfaction → Behavioral Intention (*β* = 0.229), yielding an indirect effect of 0.171 (0.748 × 0.229). The direct effect of perception on behavioral intention was *β* = 0.375 (*B* = 0.120, *p* = 0.049), which was statistically significant. The total effect was 0.546 (0.171 + 0.375). The indirect effect accounted for 31.3% (0.171/0.546) of the total effect, while the direct effect accounted for 68.7% (0.375/0.546).

Since the direct effect remained significant after accounting for the mediator (*p* = 0.049 < 0.05), the mediation is identified as partial rather than full. Model comparison using AIC indicated that the direct-effects model (AIC = 13,610) fit slightly better than the full mediation model (AIC = 13,612) (Table 8). To determine the mediating role of patient satisfaction between perception and behavioral intention, this study compared the goodness-of-fit of the full mediation model and the direct-effects model. The chi-squared difference test further showed a significant improvement of the full mediation model over the direct-effects model (Chi-square diff = 3.94, *p* = 0.047), suggesting that the mediation mechanism is meaningful. The Chi-square difference test yielded Δ*χ*^2^ = 3.9378, Δd*f* = 1, *p* < 0.05 (Table 9).

**Table 8:** Model fit indices for competing models.

Model	Df	AIC	BIC	Chi-square	RMSEA
Direct-effects model (Perception → Behavioral Intention)	61	13,610	13,758	427.22	—
Full mediation model (Perception → Satisfaction → Behavioral Intention)	62	13,612	13,755	431.16	—

**Table 9:** Chi-squared difference test results.

Comparison	Chi-square diff	Df diff	Pr (>Chi-square)	Significance
Direct-effects model vs. Full mediation model	3.9378	1	0.04721	*(*p* < 0.05)

*p < 0.05, **p < 0.01, **p < 0.001.

This indicates that patient perception influences behavioral intention through two pathways: indirectly via patient satisfaction, and directly without going through satisfaction. Therefore, H4 is supported.

Overall, the revised SEN provided a better fit to the data. The results of the hypotheses H1, H2, H3 and H4 yielded good fit indices confirming the partial mediating role of patient satisfaction (see Supplementary Table 11).

## 5 Discussion

This study analyzes the overall gap between patient expectations and perceptions regarding the quality of outpatient services in Yuhuan CIHO, which is based on the “patient-centered” concept. The overall average perceived service quality of the Yuhuan Health Community by patients was 4.19, with an expectation of 4.15 and an overall satisfaction level of 4. The results reflect a positive gap between patients’ perceptions and expectations regarding the overall quality of outpatient services. Specifically, the service quality has met patients’ expected quality. This is largely consistent with the predictions of the service quality gap model [Parasuraman et al., (42)], which suggests that patients will give positive evaluations of service quality when perceived performance exceeds expectations. As per the structural equation model, patients’ service quality expectations were a significant and positive predictor of their perceptions (*β* = 0.953). In other words, higher expectations led to higher perceptions. This also indirectly influenced their future healthcare-seeking intentions through the mediating pathway of “perception to satisfaction.”

The study by Zhou et al. showed that the establishment of a CIHO can be crucial, especially the enhancement of the quality of outpatient services in health centers (48). International experience shows that integrated care models can reduce avoidable outpatient visits, generate short-term cost savings, and improve patient satisfaction. Compared with international integrated care models, CIHO features a clearer organizational structure and tighter integration. In terms of payment mechanisms, global budgeting emphasizes shared regional risk, whereas ACOs focus more on performance-based incentives through shared savings. Levesque et al. (49) proposed an innovative integrated health performance measurement framework, and pointed out that by integrating the perspectives of patients, clinicians, and the system, the true value of the integrated healthcare system can be fully reflected. Regarding service scope, CIHO combines both public health and clinical services, while PCMH and ACO emphasize clinical coordination. As global health systems increasingly transition toward digitalization, recent studies indicate that digital health interventions can enhance system resilience and responsiveness, though patient-centered principles must be prioritized (50, 51). In terms of population coverage, CIHO operates within geographic administrative boundaries to achieve universal population coverage, whereas international models typically target insured or enrolled populations. Singapore’s Patient-Centered Medical Home (PCMH) study showed that this model improves care experiences for older adult(s), with participants reporting positive outcomes in integrated appointment scheduling and sustained doctor-patient relationships (52). Compared with typical international models, CIHO better aligns with China’s practical needs for building an integrated healthcare system, particularly in fulfilling public health functions and implementing comprehensive population health management. Offering a viable reference for low- and middle-income countries advancing healthcare integration.

The integrated development of county, township, and village healthcare service systems plays a significant role in enhancing outpatient service quality. In December 2023, the National Health Commission, Ministry of Finance and eight other departments of China jointly issued the Guidelines on Comprehensively Promoting the Construction of Close-knit County Community Healthcare, which put forward clear requirements for accelerating the construction of close-knit county community healthcare (53). In China, the current construction of close-knit county healthcare communities is manifested mainly in two models: the government-led model and the hospital-led model (54–56). Funan Healthcare Community, Anhui Province (57), a government-led model, mainly depends on the local government to upgrade the ability of primary healthcare services through policy guidance and allocation of resources. The “hospital-led” model ofthe Nanxun Second Health Community Group in Zhejiang Province utilizes the technical and managerial strengths of leading hospitals as a powerful driver to ameliorate the quality of health service for the whole Health community (58). Both models take the patient-centered approach, aiming to optimize service processes and improve service accessibility, thereby achieving or even exceeding patient expectations and creating a positive gap in terms of experience (59). The practice of the Yuhuan CIHO also demonstrates the typical characteristics of the ‘hospital-led’ model. The CIHO has simultaneously implemented corresponding reforms in information systems, payment methods, and cultural integration to enhance patient perception and satisfaction. Artificial intelligence, as an aid to clinical decision-making, can optimize processes such as appointment scheduling, triage, referrals, follow-up, and information sharing within integrated healthcare organizations, thereby improving efficiency, responsiveness, and continuity of care (60). However, the implementation of artificial intelligence requires establishing clinical governance mechanisms, maintaining human oversight, strengthening data protection, providing staff training, and monitoring system errors to ensure patient safety and clear legal accountability (61). In November 2025, the National Health Commission, along with five other ministries, promulgated the “Opinions on Deepening the Implementation of the Artificial Intelligence + Action,” which emphasizes the use of artificial intelligence technology to optimize intelligent service processes for patients, providing full-process services before, during, and after diagnosis, in order to improve the patient healthcare experience (62). Doctors can leverage AI technology and allow detailed sharing of electronic health records so that they can quickly access critical information including patients’medical histories, allergy records, and symptoms (63). These actions also offer support for the development of personalized diagnosis and treatment.

As the leading hospital of Yuhuan CIHO, Yuhuan People’s Hospital has effectively improved the environment, equipment, staff professionalism, and service attitudes of primary healthcare institutions through resource allocation, technical assistance, and unified management. This has gained patient recognition and even exceeded their initial expectations of primary healthcare. The results showed that Haishan Branch (Y3) had higher perceived than expected quality scores in assurance (*W* = 411.000, *p* = 0.007), while Kanmen Branch (Y4) scored higher in tangibles (*W* = 726.500, *p* = 0.006). However, patients’ perceptions still fall short of their expectations regarding the reliability, responsiveness, and assurance dimensions of outpatient services across different tiers of healthcare institutions within the Health Community. At Yuhuan People’s Hospital (Y1), patient perceptions of responsiveness and assurance are significantly lower than their expectations, and at Damaiyu Branch (Y2), patient perceptions of reliability are lower than their expectations (*W* = 1084.500, *p* = 0.040). These findings are in line with other studies using SERVQUAL. Reliability has become a priority for managers in healthcare institutions to enhance medical service quality: Mohammadi et al. (64) argue that, through the empowerment of the staff to ensure high-quality healthcare delivery, the reliability of medical care will be enhanced. Moreover, Bowling et al. (22) suggest that primary general practice services achieve an even greater level of patient satisfaction than outpatient services offered by hospitals in terms of continuity and doctor-patient communication. Nonetheless, various levels and types of medical institutions take on disparate functional roles within the health community (65). Primary healthcare institutions mainly offer general medical services for common and frequently encountered diseases as well as basic public health services (66). In terms of efficiency, healthcare institutions at different levels exhibit differentiated quality issues. From a health economics perspective, county hospitals should prioritize improving responsiveness and assurance, while primary-level facilities should focus on enhancing reliability, by clearly defining the functional roles of each level and unifying quality control standards. County-level hospitals, on the other hand, focus on the diagnosis and treatment of critically ill patients, medical research and education (67). The outpatients of Yuhuan People’s Hospital may have higher expectations for high-tech and complex diseases diagnosis and treatment, while that of the five branch hospitals pay more attention to the convenience, continuity of service and humanistic care. This requires the CIHO to have a clear definition of the functional positioning of the Yuhuan CIHO of all levels of the system, and guide patients to form a reasonable expectations and to reduce the expectation-perception gap caused by “mismatched medical visits.”

The results also show that the patients’ socio-demographic characteristics (gender, age, education, occupation, income, health insurance type) and healthcare-seeking behavior significantly affect expectations and perceptions of all dimensions. Male patients scored slightly higher than female patients across multiple dimensions, including tangibility (*W* = 145,515, *p* = 0.008), reliability (*W* = 146146.5, *p* = 0.005), and assurance (*W* = 144451.5, *p* = 0.015). This result is consistent with the findings of Jonkisz et al. (35). In terms of healthcare-seeking characteristics, patients enrolled in New Rural Cooperative Medical Scheme had significantly lower perceptions of “tangibles.” Patients with 6–10 visits had significantly lower scores on “responsiveness” and “empathy.” A longitudinal study on chronic disease patients in China [Fan (41)] suggests that when the number of medical visits exceeds a particular threshold, doctor-patient communication patterns start to become routine, with doctors focusing more on monitoring the medical conditions rather than providing emotional support, leading to a perception of lower service investment by patients. Yuhuan CIHO focuses on individual health services in accordance with the diverse needs of the patients and utilizes multi-dimensional strategies including health prevention and promotion, self-management support, disease management, and case management (31). The implementation of evidence-based standard operating procedures for outpatient services has helped to further standardize the clinical practice of healthcare professionals and improve service quality and patient satisfaction (68). In terms of equity, CIHO reduces disparities in service quality among institutions at different levels through resource allocation and technical support, promoting equal access to medical services within regions. An evaluation study on county-level medical alliances in Henan Province indicated that current integrated care services still fac. Constraints in funding, human resources, and information technology, and the diverse needs of vulnerable groups such as the older adult(s) and patients with chronic diseases remain inadequately addressed (69). In advancing integrated CIHO, we should focus on the differentiated needs of vulnerable populations and prioritize allocating service improvement resources to those who need them most.

The relationships among patient expectations and perceptions, patient satisfaction, and behavioral intentions were also studied. Through the assessment of the connection between expectation and perception, healthcare organizations can deliver patient-centered medical care in a more appropriate manner (35, 70). The empirical evidence also enables the health community to achieve systematic improvement of service quality through internal management and resource integration optimization. According to the modified structural equation modeling results, patient expectations influence actual perceptions (*β* = 0.953, *p* < 0.001). Patient perception was the most primary and direct factor influencing patient satisfaction. The behavioral intention of patients was significantly influenced by patient satisfaction (*β* = 0.229, *p* < 0.001). Satisfaction is an evaluation formed by patients after comparing their perceived service quality with their expectations. Previous studies have shown that perceived medical service quality significantly and positively predicts satisfaction (γ = 0.87), and perception also indirectly influences behavioral intentions through satisfaction (71). In this study, the standardized path coefficient of perception on satisfaction reached 0.748 (*p* < 0.001), further confirming the applicability of this relationship within the CIHO context. Regarding sustainability, the positive effect of patient satisfaction on behavioral intentions (*β* = 0.229, *p* < 0.001) indicates that satisfied patients are more likely to remain within the system for future care or recommend it to others. These findings are consistent with the study by Amarantou et al. (72), with patient satisfaction being a significant mediator of the relationship between patients’ perceived service quality and other behavioral intentions. When choosing a healthcare facility, patients consider a variety of factors, including the severity of their condition, convenience of access, and insurance reimbursement policies, among others (73). The quality of outpatient service provided must meet or even exceed the expectations and needs of the patients. This often helps to attract and retain outpatients (74). When patients’ actual needs and demands are met by healthcare services, they are more likely to return for future care. Further, they will also refer others to the facility, thus helping facilities attract more patients (75). To meet the desired outcomes, healthcare providers are required to pay attention to the patient expectations and implement effective services while delivering high-quality care (76). The conclusions of relevant research literature also indicate that when patients perceive personalized and professional medical services, they have a positive evaluation of the services, which helps enhance their commitment to medical institutions (77–79). Accordingly, the continuous optimization of outpatient service procedures, improvement in the quality of outpatient services, and enhancement of patients’ healthcare experiences are important for improving the outpatient services and competitiveness of Yuhuan CIHO. According to the analysis, this model encourages patient satisfaction and the improvement of outpatient medical services (80).

## 6 Conclusion

This study evaluates the outpatient service quality of the Yuhuan CIHO from a patient-centered perspective. The results indicate that healthcare institutions within the Yuhuan CIHO provide a positive patient experience in terms of service environment, facility conditions, and the professionalism of medical staff, with actual patient perceptions meeting or even exceeding their expectations. However, there is an expectation-perception gap regarding “responsiveness” and “assurance” at the county-level hospital, and a similar gap regarding “reliability” at the primary care institution Y2. The structural equation modeling further reveals that patients’ perceptions of outpatient service quality significantly influence their satisfaction, with patient satisfaction serving as a mediating variable that notably affects their healthcare-seeking behavioral intentions. This reflects the need for healthcare providers to focus on patient expectations and perceptions in order to optimize the healthcare experience. The study emphasizes that within the county-level health community framework, improving outpatient service quality is not the task of a single institution. The enhancement of service quality faces deep challenges, transitioning from “resource coverage” to “refined management,” requiring a multidimensional evaluation of functional contradictions at different levels of institutions and attention to patient expectations and experiences. By leveraging information technology to strengthen coordination, county-level hospitals need to optimize processes and manage patient expectations regarding response speed, while primary care institutions need to further improve reliability to bridge service gaps. It is recommended that CIHO adopt differentiated improvement strategies based on the specific shortcomings of county-level hospitals and primary healthcare institutions, incorporate patient experience monitoring into performance evaluation systems, and establish a closed-loop management mechanism of “measurement—feedback—improvement.”

Several limitations should be acknowledged. First, this study employed a cross-sectional design with all variables measured at the same time point using self-reported questionnaires, which introduces the potential for common method bias and precludes causal inferences. Second, the purposive sampling of outpatients from a single CIHO—although yielding a sample of 1,033 respondents across six institutions—limits the generalizability of our findings to other regions, healthcare delivery models, or temporal settings. The standardized coefficient between expectation and perception approached unity (*β* = 0.953), suggesting that patients may not have clearly distinguished between their ex ante expectations and ex post perceptions when completing the questionnaire. This may indicate that respondents tended to provide similar ratings for the two constructs, regardless of their theoretical distinctiveness.

Future research should refine the measurement of these dimensions, possibly by using more distinct item wordings or objective indicators. Longitudinal designs, comparative studies across other county-level integrated health systems, and incorporation of objective clinical performance indicators are also encouraged to further validate and extend our findings.

## Data Availability

The original contributions presented in the study are included in the article/Supplementary material, further inquiries can be directed to the corresponding authors.
