# Correction: A cross-sectional study on patient-centered outpatient service quality in the county-level integrated health organization of Yuhuan, Zhejiang province of China

**DOI:** 10.3389/fpubh.2026.2019456

**Published:** 2026-10-19

**Authors:** Qin Wang, Qihao Shen, Liming Shao, Qing Xu, Yijing Xie

**Affiliations:** 1 School of Public Health, Hangzhou Medical College, Hangzhou, Zhejiang, China; 2 School of Medical Humanities and Management (School of General Practice Competence Education), Hangzhou Medical College, Hangzhou, Zhejiang, China; 3 School of Population Medicine and Public Health, Chinese Academy of Medical Sciences and Peking Union Medical College, Beijing, China; 4 School of Education, Central China Normal University, Hangzhou, Zhejiang, China; 5 Institute of Health Policy, Hangzhou Medical College, Hangzhou, Zhejiang, China

**Keywords:** mediation effect, outpatient service quality, patient expectation and perception, patient intention to seek medical treatment, patient-centered, the county-level integrated health organization

In the **Funding** statement, the sentence “We appreciate the support from Key Discipline of Zhejiang Province in Public Health and Preventive Medicine (First Class, Category A), Hangzhou Medical College.” was erroneously omitted and needs to be supplemented, as this project covered the article publication cost.

The correct **Funding** statement is: “The author(s) declared that financial support was received for this work and/or its publication. This work was funded through Zhejiang Kangenbei Hospital Management Soft Science Research Project (2024ZHA-KEB322), fund of the Basic Research Program of Hangzhou Medical College for Basic Scientific Research Project (KYZD2024007), Open Project of the China (Guangxi)—ASEAN Research Center for Modern Governance of Public Health Security in 2025 (CAMGCPHS202512). We appreciate the support from Key Discipline of Zhejiang Province in Public Health and Preventive Medicine (First Class, Category A), Hangzhou Medical College.”

In the **Acknowledgments**, the sentence “We appreciate the support from Key Discipline of Zhejiang Province in Public Health and Preventive Medicine (First Class, Category A), Hangzhou Medical College. This project covered the article publication cost.” was erronenously omitted and needs to be supplemented.

The correct **Acknowledgments** statement is: “The authors are grateful to the County-level Integrated Health Organization (CIHO) of Yuhuan People's Hospital (Yuhuan First CIHO) and Hangzhou Medical College. We extend our heartfelt gratitude to all the participants in our study for their time and valuable collaboration. We appreciate the support from Key Discipline of Zhejiang Province in Public Health and Preventive Medicine (First Class, Category A), Hangzhou Medical College. This project covered the article publication cost.”

The original version of this article has been updated.

