# Supplementary material for: A cross-sectional study on patient-centered outpatient service quality in the county-level integrated health organization of Yuhuan, Zhejiang province of China
Source: Front Public Health. 2026 Sep 23;14:1889257. doi: 10.3389/fpubh.2026.1889257 (PMC13642747; doi:10.3389/fpubh.2026.1889257)
Supplement: Supplementary file 1 [file Supplementary_file_1.docx]

**Supplementary Table 1** Reliability Analysis Results of the Questionnaire

| Variable Type | Variable Name | Number of Items | Cronbach's α |
| --- | --- | --- | --- |
| Total Scale | Expectation Total Scale | **23** | **0.981** |
|  | Perception Total Scale | **23** | **0.980** |
| Expectation Scale | Tangibles | 5 | 0.930 |
|  | Reliability | 5 | 0.906 |
|  | Responsiveness | 5 | 0.925 |
|  | Assurance | 4 | 0.920 |
|  | Empathy | 4 | 0.859 |
| Perception Scale | Tangibles | 5 | 0.916 |
|  | Reliability | 5 | 0.922 |
|  | Responsiveness | 5 | 0.929 |
|  | Assurance | 4 | 0.908 |
|  | Empathy | 4 | 0.829 |

**Supplementary Table 2** Results of KMO and Bartlett’s Test of Sphericity

| Test Indicator | Value | Judgment Criteria |
| --- | --- | --- |
| Kaiser-Meyer-Olkin Measure of Sampling Adequacy | 0.947 | >0.90 indicates excellent |
| Bartlett's Test of Sphericity | 3523.420 | p<0.05 |
| df | 253 |  |
| Sig. | 0.000 |  |

**Supplementary Table 3** Total Variance Explained Table

| Component | Initial Eigenvalues | | | Extraction Sums of Squared Loadings | | | Rotation Sums of Squared Loadings | | |
| --- | --- | --- | --- | --- | --- | --- | --- | --- | --- |
|  | Total | Variance% | Cumulative % | Total | Variance% | Cumulative % | Total | Variance% | Cumulative % |
| 1 | 16.185 | 70.368 | 70.368 | 16.185 | 70.368 | 70.368 | 6.138 | 26.688 | 26.688 |
| 2 | 0.803 | 3.490 | 73.858 | 0.803 | 3.490 | 73.858 | 4.598 | 19.993 | 46.681 |
| 3 | 0.723 | 3.143 | 77.001 | 0.723 | 3.143 | 77.001 | 3.504 | 15.233 | 61.915 |
| 4 | 0.642 | 2.792 | 79.793 | 0.642 | 2.792 | 79.793 | 2.626 | 11.416 | 73.330 |
| 5 | 0.501 | 2.180 | 81.973 | 0.501 | 2.180 | 81.973 | 1.988 | 8.642 | 81.973 |

**Notes:**

**Abbreviations:** Only factors with eigenvalues greater than 1 are displayed; subsequent factors were not retained due to eigenvalues less than 1.

**Supplementary Table 4** Results of KMO and Bartlett’s Test of Sphericity

| Test Indicator | Value | Judgment Criteria |
| --- | --- | --- |
| Kaiser-Meyer-Olkin Measure of Sampling Adequacy | 0.950 | >0.90 indicates excellent |
| Bartlett’s Test of Sphericity | 3461.232 | p < 0.05 |
| df | 253 |  |
| Sig. | 0.000 |  |

**Supplementary Table 5** Total Variance Explained Table

| Component | Initial Eigenvalues | | | Extraction Sums of Squared Loadings | | | Rotation Sums of Squared Loadings | | |
| --- | --- | --- | --- | --- | --- | --- | --- | --- | --- |
|  | Total | Variance% | Cumulative % | Total | Variance% | Cumulative % | Total | Variance% | Cumulative % |
| 1 | 16.049 | 69.778 | 69.778 | 16.049 | 69.778 | 69.778 | 5.662 | 24.619 | 24.619 |
| 2 | .937 | 4.075 | 73.854 | .937 | 4.075 | 73.854 | 4.139 | 17.997 | 42.616 |
| 3 | .646 | 2.807 | 76.661 | .646 | 2.807 | 76.661 | 3.693 | 16.057 | 58.673 |
| 4 | .588 | 2.557 | 79.217 | .588 | 2.557 | 79.217 | 3.279 | 14.258 | 72.931 |
| 5 | .527 | 2.291 | 81.509 | .527 | 2.291 | 81.509 | 1.973 | 8.578 | 81.509 |

**Notes:**

**Abbreviations:** Only factors with eigenvalues greater than 1 are displayed; subsequent factors were not retained due to eigenvalues less than 1.

**Supplementary Table 6** Tests for Differences Across Demographic Variables

|  | N | eTangibles | eReliability | eResponsiveness | eAssurance | eEmpathy | pTangibles | pReliability | pResponsiveness | pAssurance | pEmpathy |
| --- | --- | --- | --- | --- | --- | --- | --- | --- | --- | --- | --- |
|  |  | Median (Q1,Q3) | Median (Q1,Q3) | Median  (Q1,Q3) | Median (Q1,Q3) | Median (Q1,Q3) | Median  (Q1,Q3) | Median  (Q1,Q3) | Median  (Q1,Q3) | Median (Q1,Q3) | Median  (Q1,Q3) |
| **Age** | | | | | | | | | | | |
| <40 | 504 | 4  (3.8, 4.6) | 4  (3.6, 4.6) | 4  (3.8, 4.6) | 4  (3.75, 4.75) | 4  (3.75, 4.75) | 4  (3.8, 4.6) | 4  (3.6, 4.6) | 4  (3.6, 4.6) | 4  (3.5, 4.75) | 4  (3.75, 4.75) |
| 40-59 | 336 | 4  (3.8, 5) | 4  (3.8, 5) | 4  (3.6, 5) | 4  (3.75, 5) | 4.25  (3.75, 5) | 4  (3.8, 5) | 4  (3.75, 5) | 4  (3.8, 5) | 4  (3.75, 5) | 4  (3.75, 5) |
| >60 | 189 | 4  (3.6, 5) | 4.2  (3.8, 5) | 4  (3.4, 5) | 4.25  (4, 5) | 4.5  (4, 5) | 4.2  (4, 5) | 4.2  (3.8, 5) | 4.2  (3.8, 5) | 4.5  (3.75, 5) | 4.75  (3.75, 5) |
| *H* |  | 7.592 | 8.137 | 5.223 | 10.949 | 19.649 | 6.367 | 5.791 | 9.697 | 12.491 | 10.543 |
| *P* |  | 0.022^*^ | 0.017^*^ | 0.073 | 0.004^**^ | < 0.001^***^ | 0.041^*^ | 0.055 | 0.008^**^ | 0.002^**^ | 0.005^**^ |
| **Sex** | | | | | | | | | | | |
| Male | 490 | 4.2  (3.8, 5) | 4.2  (3.8, 5) | 4  (3.8, 5) | 4.25  (3.75, 5) | 4  (3.75, 5) | 4.2  (4, 5) | 4.2  (3.8, 5) | 4.2  (3.8, 5) | 4.25  (3.75, 5) | 4.25  (3.75, 5) |
| Female | 543 | 4  (3.6, 4.8) | 4  (3.6, 4.8) | 4  (3.6, 4.8) | 4  (3.5, 5) | 4  (3.75, 5) | 4  (3.6, 5) | 4  (3.6, 5) | 4  (3.6, 5) | 4  (3.5, 5) | 4  (3.75, 5) |
| *W* |  | 145515 | 146146.5 | 142121 | 144451.5 | 140460 | 147915 | 144693 | 142097 | 141883 | 145370 |
| *P* |  | 0.008^**^ | 0.005^**^ | 0.054 | 0.015^*^ | 0.112 | 0.002^**^ | 0.013^*^ | 0.055 | 0.06 | 0.008^**^ |
| **Education** | | | | | | | | | | | |
| Primary school or below | 245 | 4.6  (4, 5) | 4.6  (4, 5) | 4.4  (4, 5) | 4.75  (4, 5) | 5  (4, 5) | 4.4  (4, 5) | 4.6  (4, 5) | 4.6  (4, 5) | 4.75  (4, 5) | 5  (4, 5) |
| Middle school | 406 | 4  (3, 4.4) | 4  (3, 4.6) | 4  (3.2, 4.6) | 4  (3, 4.75) | 4  (3.25, 4.75) | 4  (3, 4.4) | 4  (3.05, 4.4) | 4  (3, 4.4) | 4  (3, 4.5) | 4  (3.25, 4.75) |
| College or above | 322 | 4.1  (4, 5) | 4  (4, 5) | 4.1  (4, 5) | 4  (4, 5) | 4.12  (4, 5) | 4.2  (4, 5) | 4.2  (4, 5) | 4  (3.85, 5) | 4  (4, 5) | 4.25  (4, 5) |
| *H* |  | 63.378 | 59.448 | 64.586 | 57.071 | 62.614 | 68.109 | 59.039 | 66.801 | 73.237 | 71.677 |
| *P* |  | < 0.001^***^ | < 0.001^***^ | < 0.001^***^ | < 0.001^***^ | < 0.001^***^ | < 0.001^***^ | < 0.001^***^ | < 0.001^***^ | < 0.001^***^ | < 0.001^***^ |
| **Job** | | | | | | | | | | | |
| Famer | 102 | 4.4  (4, 5) | 4.4  (4, 5) | 4.4  (3.65, 5) | 4.5  (3.81, 5) | 4.75  (4, 5) | 4.4  (3.85, 5) | 4.3  (4, 5) | 4.4  (4, 5) | 4.5  (4, 5) | 5  (4, 5) |
| P&T Equip. Ops. & Assoc. Staff | 105 | 4.6  (3.2, 5) | 4.6  (3, 5) | 4.4  (3.2, 5) | 4.75  (3, 5) | 4.75  (3.25, 5) | 4  (3, 5) | 4  (3, 5) | 4.2  (3, 5) | 4  (3, 5) | 4  (3.25, 5) |
| Bus. & Serv. Staff | 229 | 4  (3.6, 5) | 4  (3.6, 5) | 4  (3.4, 5) | 4  (3.25, 5) | 4  (3.5, 5) | 4  (3.6, 5) | 4  (3.6, 5) | 4  (3.4, 5) | 4  (3.25, 5) | 4  (3.5, 5) |
| Admin. Staff | 32 | 4.6  (4, 5) | 4.4  (4, 5) | 4.6  (4, 5) | 4.62  (4, 5) | 4.75  (4, 5) | 4.7  (4, 5) | 4.4  (4, 5) | 4.6  (4, 5) | 4.5  (4, 5) | 4.5  (3.94, 5) |
| Ent. Staff | 110 | 4  (3.6, 4.55) | 4  (3.45, 4.6) | 4  (3.45, 4.6) | 4  (3.75, 4.75) | 4  (3.5, 4.5) | 4  (3.4, 4.4) | 4  (3.4, 4.6) | 4  (3.25, 4.6) | 4  (3.25, 4.69) | 4  (3.25, 4.75) |
| Prof. Tech. Staff | 89 | 4  (4, 5) | 4  (4, 5) | 4  (4, 5) | 4.25  (4, 5) | 4.25  (4, 5) | 4  (4, 5) | 4.2  (4, 5) | 4  (4, 5) | 4  (4, 5) | 4.25  (4, 5) |
| Soldier | 10 | 2.7 (2.4,3.5) | 3.1  (2.5, 4.4) | 2.8  (2.6, 4.05) | 2.8  (2.5, 4.5) | 3  (2.5, 4) | 2.9  (2.4, 4.1) | 2.9  (2.6, 3.9) | 2.9  (2.6, 4.3) | 2.3  (2.2, 3.9) | 2.9  (2.5, 3.4) |
| Student | 172 | 4.2  (4, 4.6) | 4.2 (4, 4.6) | 4.2  (4, 4.45) | 4.25  (4, 4.5) | 4.1  (3.9, 4.5) | 4.2  (4, 4.6) | 4.2  (4, 4.45) | 4.2  (4, 4.4) | 4.25  (4, 4.5) | 4.25  (4, 4.5) |
| Retire | 104 | 3.7  (2.8, 4.4) | 3.8  (2.8, 4.2) | 3.6  (2.8, 4.2) | 4  (2.75, 4.5) | 3.9  (2.7, 4.3) | 3.8  (2.6, 4.2) | 3.6  (2.8, 4.2) | 3.8  (2.8, 4.4) | 3.7  (2.7, 4.5) | 3.7  (2.7, 4.2) |
| Unemp. | 80 | 4.3  (4, 5) | 4.4  (4, 5) | 4.4  (4, 5) | 4.5  (4, 5) | 4.88  (4, 5) | 4.2  (3.95, 5) | 4.6  (4, 5) | 4.3  (3.8, 5) | 4.5  (3.75, 5) | 4.75  (4, 5) |
| *H* |  | 65.22 | 59.278 | 67.05 | 51.42 | 71.156 | 52.332 | 58.583 | 45.225 | 55.952 | 69.469 |
| *P* |  | < 0.001^***^ | < 0.001^***^ | < 0.001^***^ | < 0.001^***^ | < 0.001^***^ | < 0.001^***^ | < 0.001^***^ | < 0.001^***^ | < 0.001^***^ | < 0.001^***^ |
| **Income** | | | | | | | | | | | |
| <2000RMB | 245 | 4.2 (4, 4.8) | 4.2 (4, 4.8) | 4 (3.8, 4.8) | 4.25 (4, 5) | 4 (3.75, 5) | 4.2 (4, 4.6) | 4.2 (4, 4.8) | 4.2 (4, 4.8) | 4.25 (4, 5) | 4.25 (4, 5) |
| 2000~5000  RMB | 377 | 4  (3.2, 4.8) | 4  (3.2, 4.8) | 4  (3.2, 4.6) | 4  (3.25, 4.75) | 4  (3.5, 5) | 4  (3.4, 4.8) | 4  (3.2, 4.8) | 4  (3.2, 4.6) | 4  (3.25, 5) | 4  (3.5, 5) |
| 5000~10000  RMB | 356 | 4  (3.8, 5) | 4  (3.8, 5) | 4 (3.8, 5) | 4 (3.75, 5) | 4.25  (4, 5) | 4.2  (3.8, 5) | 4.2  (3.8, 5) | 4  (3.6, 5) | 4  (3.75, 5) | 4.25  (3.75, 5) |
| >10000RMB | 55 | 4.2  (4, 4.6) | 4.2  (3.9, 4.6) | 4.2 (4, 4.5) | 4.2 (3.7, 4.5) | 4.2  (3.7, 4.5) | 4.2  (4, 4.7) | 4.2  (3.8, 4.6) | 4  (3.7, 4.5) | 4  (3.75, 4.62) | 4  (3.75, 4.75) |
| *H* |  | 15.957 | 19.212 | 15.702 | 14.541 | 16.08 | 15.163 | 17.763 | 14.888 | 12.192 | 17.378 |
| *P* |  | 0.001 | < 0.001 | 0.001 | 0.002 | 0.001 | 0.002 | < 0.001 | 0.002 | 0.007 | < 0.001 |
| **Medical insurance** | | | | | | | | | | | |
| URBMI | 424 | 4.2  (3.6, 5) | 4.2  (3.6, 5) | 4.2  (3.6, 5) | 4.25  (3.75, 5) | 4.25  (3.75, 5) | 4.2  (3.8, 5) | 4  (3.6, 5) | 4  (3.6, 5) | 4  (3.75, 5) | 4.12  (3.75, 5) |
| UWM | 234 | 4  (3.2, 4) | 4  (3, 4) | 4  (3.2, 4.15) | 4  (3, 4) | 4  (3.25, 4) | 3.9  (3, 4.2) | 3.8  (3, 4.2) | 3.8  (3, 4) | 3.75  (3, 4.25) | 3.9  (3.2, 4.2) |
| NCMS | 200 | 4.4  (4, 5) | 4.4  (4, 5) | 4.4  (4, 5) | 4.75  (4, 5) | 5  (4, 5) | 4.6  (4, 5) | 4.6  (4, 5) | 4.6  (4, 5) | 4.75  (4, 5) | 5  (4, 5) |
| CI | 91 | 4  (3.3, 4.4) | 4  (3, 4.4) | 4  (3, 4.4) | 4  (3.12, 4.5) | 4  (3.25, 4.5) | 4  (3.2, 4.4) | 4  (3.2, 4.5) | 4  (3, 4.4) | 4  (3, 4.25) | 4  (3.12, 4.38) |
| SMI | 84 | 4.2  (4, 4.6) | 4.2  (4, 4.6) | 4.3  (4, 4.6) | 4.25  (4, 4.5) | 4.25  (4, 4.75) | 4.3  (4, 4.6) | 4.2  (4, 4.6) | 4.2  (4, 4.6) | 4.25  (4, 4.5) | 4.25  (4, 4.56) |
| *H* |  | 100.792 | 104.851 | 94.605 | 115.618 | 126.254 | 120.314 | 129.993 | 121.919 | 138.505 | 151.965 |
| *P* |  | < 0.001 | < 0.001 | < 0.001 | < 0.001 | < 0.001 | < 0.001 | < 0.001 | < 0.001 | < 0.001 | < 0.001 |
| **Household registration type** | | | | | | | | | | | |
| Rural | 562 | 4  (3.8, 4.8) | 4  (3.8, 4.8) | 4  (3.6, 4.6) | 4  (3.75, 5) | 4  (3.75, 5) | 4  (3.8, 5) | 4  (3.8, 5) | 4  (3.6, 5) | 4  (3.75, 5) | 4.25  (3.75, 5) |
| Urban | 471 | 4  (3.8, 5) | 4  (3.6, 5) | 4  (3.8, 5) | 4  (3.75, 5) | 4  (3.75, 5) | 4 (3.8, 5) | 4 (3.6, 5) | 4  (3.6, 5) | 4  (3.75, 5) | 4  (3.75, 5) |
| *W* |  | 127276 | 128175 | 123015 | 125661.5 | 129971 | 134714 | 134782.5 | 133548.5 | 133662 | 137457.5 |
| *P* |  | 0.280 | 0.374 | 0.047 | 0.153 | 0.610 | 0.615 | 0.605 | 0.799 | 0.780 | 0.274 |
| **Department** | | | | | | | | | | | |
| Internal | 195 | 5  (4, 5) | 5  (4, 5) | 5  (4, 5) | 5  (4, 5) | 5  (4, 5) | 4.8  (4, 5) | 4.8  (4, 5) | 4.6  (4, 5) | 5  (4, 5) | 5  (4, 5) |
| Surgery | 206 | 4  (3.8, 4.4) | 4  (3.65, 4.4) | 4  (3.6, 4.4) | 4  (3.75, 4.5) | 4  (3.75, 4.5) | 4  (3.6, 4.4) | 4  (3.6, 4.55) | 4  (3.6, 4.4) | 4  (3.7, 4.4) | 4  (3.75, 4.5) |
| General | 632 | 4  (3.6, 4.8) | 4  (3.6, 4.6) | 4  (3.4, 4.6) | 4  (3.5, 4.75) | 4  (3.69, 5) | 4  (3.6, 4.85) | 4  (3.6, 4.8) | 4  (3.6, 4.8) | 4  (3.5, 5) | 4  (3.75, 5) |
| *H* |  | 72.286 | 71.286 | 78.213 | 52.497 | 73.549 | 39.413 | 34.695 | 31.7 | 35.351 | 35.354 |
| *P* |  | < 0.001 | < 0.001 | < 0.001 | < 0.001 | < 0.001 | < 0.001 | < 0.001 | < 0.001 | < 0.001 | < 0.001 |
| **Visits** | | | | | | | | | | | |
| 1~5 | 560 | 4.1  (4, 5) | 4  (3.8, 5) | 4  (3.8, 5) | 4  (3.75, 5) | 4  (4, 5) | 4  (4, 5) | 4  (3.8, 5) | 4  (3.8, 5) | 4  (3.75, 5) | 4  (3.75, 5) |
| 6~10 | 210 | 4.2  (3.8, 4.8) | 4.2  (3.8, 4.8) | 4.2  (3.8, 4.6) | 4.25  (4, 4.9) | 4.25  (3.7, 5) | 4.2  (3.8, 4.8) | 4.2  (3.8, 4.8) | 4.2  (3.8, 4.8) | 4.25  (3.7, 5) | 4.25  (3.75, 5) |
| 11~15 | 175 | 4  (3.6, 5) | 4  (3.4, 5) | 4  (3.6, 5) | 4.25  (3.5, 5) | 4  (3.5, 5) | 4  (3.6, 5) | 4.2  (3.6, 4.9) | 4  (3.6, 5) | 4.25  (3.6, 5) | 4.25  (3.75, 5) |
| 16+ | 88 | 3.3  (2.55, 4) | 3  (2.6, 4.2) | 3.2  (2.6, 4.2) | 3  (2.5, 4.31) | 3.4  (2.7, 4.3) | 3.2  (2.4, 4.2) | 3.2  (2.6, 4.4) | 3  (2.6, 4.4) | 3.1  (2.5, 4.3) | 3.2  (2.5, 4.5) |
| *H* |  | 55.915 | 39.254 | 39.143 | 43.879 | 36.449 | 39.459 | 30.665 | 41.909 | 36.650 | 35.389 |
| *P* |  | < 0.001 | < 0.001 | < 0.001 | < 0.001 | < 0.001 | < 0.001 | < 0.001 | < 0.001 | < 0.001 | < 0.001 |
| **Hospital** | | | | | | | | | | | |
| Y1 | 522 | 4  (3.6, 5) | 4  (3.4, 5) | 4  (3.4, 5) | 4  (3.5, 5) | 4  (3.5, 5) | 4  (3.6, 5) | 4  (3.4, 5) | 4  (3.4, 5) | 4  (3.25, 5) | 4  (3.5, 5) |
| Y2 | 79 | 4.2  (4, 4.6) | 4  (4, 4.6) | 4  (3.9, 4.4) | 4  (3.75, 4.5) | 4  (3.7, 4.4) | 4.2  (3.6, 4.4) | 4  (3.6, 4.6) | 4  (3.8, 4.4) | 4  (3.7, 4.2) | 4.2  (3.7, 4.5) |
| Y3 | 132 | 4.6  (4.15, 5) | 4.4  (4, 4.8) | 4.4  (4, 5) | 4.75  (4, 5) | 4.75  (4, 5) | 4.6  (4, 5) | 4.6  (4, 5) | 4.6  (4, 5) | 4.7  (4.25, 5) | 5  (4, 5) |
| Y4 | 120 | 4  (3.75, 4.4) | 4  (3.8, 4.4) | 4  (3.6, 4.45) | 4  (3.9, 4.5) | 4  (4, 4.56) | 4  (4, 4.6) | 4  (3.8, 4.6) | 4  (3.8, 4.6) | 4  (3.7, 4.7) | 4  (4, 4.75) |
| Y5 | 92 | 4.1  (4, 4.6) | 4.2  (3.6, 4.8) | 4.2  (3.8, 4.6) | 4.2  (3.7, 4.8) | 4  (3.69, 5) | 4.1  (3.8, 4.8) | 4.2  (3.95, 5) | 4.2  (3.8, 5) | 4.25  (3.5, 5) | 4  (3.75, 5) |
| Y6 | 88 | 4.2  (3.8, 4.6) | 4.2  (3.8, 4.6) | 4  (3.8, 4.6) | 4.2  (3.7, 4.5) | 4.2  (3.7, 4.7) | 4.2  (3.9, 4.6) | 4.2  (3.8, 4.6) | 4.2  (3.8, 4.6) | 4.2  (3.7, 4.7) | 4.2  (3.7, 4.7) |
| *H* |  | 22.248 | 16.095 | 17.439 | 19.725 | 30.774 | 27.078 | 26.512 | 27.726 | 40.202 | 32.336 |
| *P* |  | < 0.001 | 0.007 | 0.004 | 0.001 | < 0.001 | < 0.001 | < 0.001 | < 0.001 | < 0.001 | < 0.001 |

**Notes:**

**Abbreviations:** *P < 0.05, **P < 0.01, ***P < 0.001,P&T Equip. Ops. & Assoc. Staff：Production, Transportation & Equipment Operators, and Associate Staff,Bus. & Serv. Staff：Business & Service Staff,Admin. Staff：Administrative Staff,Ent. Staff：Entertainment Staff,Prof. Tech. Staff：Professional & Technical Staff,URBMI：Urban and Rural Residents Basic Medical Insurance,UWM：Urban Employee Basic Medical Insurance,NCMS：New Rural Cooperative Medical System,CI：Commercial Insurance,SMI：Student Medical Insurance,Y1 : Yuhuan People’s Hospital , Y2 : Damaiyu THCs , Y3 : Haishan THCs , Y4 : Kanmen THCs , Y5 : Lupu THCs , Y6 : Yucheng THCs .

**Supplementary Table 7** Wilcoxon Signed-Rank Test of Expectations and Perceptions

|  | Y1 | Y2 | Y3 | Y4 | Y5 | Y6 |
| --- | --- | --- | --- | --- | --- | --- |
|  | *N* = 522 | *N* = 79 | *N* = 132 | *N* = 120 | *N* = 92 | *N* = 88 |
| ETangibles  Median (Q1,Q3) | 4  (3.6, 5) | 4.2  (4, 4.6) | 4.6  (4.15, 5) | 4  (3.75, 4.4) | 4.1  (4, 4.6) | 4.2  (3.8, 4.6) |
| PTangibles  Median (Q1,Q3) | 4  (3.6, 5) | 4.2  (3.6, 4.4) | 4.6  (4, 5) | 4  (4, 4.6) | 4.1  (3.8, 4.85) | 4.2  (3.95, 4.6) |
| *W* | 18609.500 | 897.500 | 938.000 | 726.500 | 754.000 | 808.000 |
| *P* | 0.141 | 0.183 | 0.283 | 0.006^**^ | 0.924 | 0.414 |
| EReliability  Median (Q1,Q3) | 4  (3.4, 5) | 4  (4, 4.6) | 4.4  (4, 4.8) | 4  (3.8, 4.4) | 4.2  (3.6, 4.8) | 4.2  (3.8, 4.6) |
| PReliability  Median (Q1,Q3) | 4  (3.4, 5) | 4  (3.6, 4.6) | 4.6  (4, 5) | 4  (3.8, 4.6) | 4.2  (3.95, 5) | 4.2  (3.8, 4.6) |
| *W* | 18676.500 | 1084.500 | 850.000 | 846.000 | 633.000 | 749.500 |
| *P* | 0.339 | 0.040^*^ | 0.202 | 0.096 | 0.178 | 0.413 |
| eResponsiveness  Median (Q1,Q3) | 4  (3.4, 5) | 4  (3.9, 4.4) | 4.4  (4, 5) | 4  (3.6, 4.45) | 4.2  (3.8, 4.6) | 4  (3.8, 4.6) |
| pResponsiveness  Median (Q1,Q3) | 4  (3.4, 5) | 4  (3.8, 4.4) | 4.6  (4, 5) | 4  (3.6, 4.6) | 4.2  (3.8, 5) | 4.2  (3.8, 4.6) |
| *W* | 20725.500 | 852.500 | 723.000 | 932.000 | 855.000 | 758.000 |
| *P* | 0.002^**^ | 0.345 | 0.075 | 0.267 | 0.660 | 0.248 |
| eAssurance  Median (Q1,Q3) | 4  (3.5, 5) | 4  (3.75, 4.5) | 4.75  (4, 5) | 4  (3.94, 4.56) | 4.25  (3.75, 4.81) | 4.25  (3.75, 4.5) |
| pAssurance  Median (Q1,Q3) | 4  (3.25, 5) | 4  (3.75, 4.25) | 4.75  (4.25, 5) | 4  (3.75, 4.75) | 4.25  (3.5, 5) | 4.25  (3.75, 4.75) |
| *W* | 16725.000 | 871.500 | 411.000 | 888.000 | 1039.000 | 765.000 |
| *P* | 0.034^*^ | 0.721 | 0.007^**^ | 0.844 | 0.242 | 0.970 |
| eEmpathy  Median (Q1,Q3) | 4  (3.5, 5) | 4  (3.75, 4.38) | 4.75  (4, 5) | 4  (4, 4.56) | 4  (3.69, 5) | 4.25  (3.75, 4.75) |
| pEmpathy  Median (Q1,Q3) | 4  (3.5, 5) | 4.25  (3.75, 4.5) | 5  (4, 5) | 4  (4, 4.75) | 4  (3.75, 5) | 4.25  (3.75, 4.75) |
| *W* | 16473.000 | 598.000 | 550.000 | 921.000 | 681.500 | 702.500 |
| *P* | 0.643 | 0.100 | 0.202 | 0.550 | 0.456 | 0.731 |

**Notes:**

**Abbreviations:** *P < 0.05, **P < 0.01, ***P < 0.001.

**Supplementary Table 8** Predictors of Patient Perception in Outpatient Service Quality

| Value | Item | *N* | *M ± SD* | *β* | 95% CI | *P* |
| --- | --- | --- | --- | --- | --- | --- |
| pTangibles | | | | | | |
| Age | <40 | 504 | 4.0 ± 0.9 |  | | |
|  | 40-59 | 336 | 4.1 ± 0.9 | −0.01 | [−0.09, 0.06] | 0.685 |
|  | >60 | 189 | 4.2 ± 0.9 | 0.13 | [0.02, 0.23] | 0.024* |
| Job | Admin. Staff | 32 | 4.2 ± 0.9 |  | | |
|  | Bus. & Serv. Staff | 229 | 3.9 ± 1.2 | −0.07 | [−0.20, 0.06] | 0.313 |
|  | Ent. Staff | 110 | 4.1 ± 0.9 | 0.13 | [0.02, 0.25] | 0.021* |
|  | Famer | 102 | 4.4 ± 0.7 | 0.11 | [−0.08, 0.30] | 0.239 |
|  | P&T Equip. Ops. & Assoc. Staff | 105 | 3.9 ± 0.9 | 0.01 | [−0.12, 0.15] | 0.857 |
|  | Prof. Tech. Staff | 89 | 4.3 ± 0.6 | 0.14 | [−0.01, 0.28] | 0.062 |
|  | Retire | 104 | 3.2 ± 1.1 | 0.11 | [−0.19, 0.41] | 0.469 |
|  | Soldier | 10 | 4.3 ± 0.4 | 0.08 | [−0.06, 0.23] | 0.270 |
|  | Student | 172 | 3.6 ± 0.9 | −0.06 | [−0.19, 0.07] | 0.384 |
|  | Unemp. | 80 | 4.2 ± 0.9 | −0.05 | [−0.18, 0.08] | 0.467 |
| Medical insurance | CI | 91 | 4.1 ± 1.0 |  | | |
|  | NCMS | 200 | 3.7 ± 0.8 | −0.13 | [−0.20, −0.05] | 0.001** |
|  | SMI | 84 | 4.4 ± 0.6 | 0.08 | [−0.02, 0.17] | 0.106 |
|  | URBMI | 424 | 3.8 ± 0.9 | −0.00 | [−0.11, 0.10] | 0.928 |
|  | UWM | 234 | 4.3 ± 0.5 | 0.01 | [−0.13, 0.16] | 0.860 |
| Household register | Rural | 562 | 4.1 ± 0.9 |  | | |
|  | Urban | 471 | 4.1 ± 0.8 | −0.05 | [−0.10, 0.01] | 0.123 |
| Visits | 1-5 | 560 | 4.2 ± 0.8 |  | | |
|  | 11-15 | 175 | 4.1 ± 0.8 | −0.02 | [−0.09, 0.06] | 0.650 |
|  | 16+ | 88 | 4.0 ± 0.9 | −0.08 | [−0.16, 0.00] | 0.057 |
|  | 6-10 | 210 | 3.4 ± 1.1 | −0.11 | [−0.22, −0.01] | 0.038* |
| eTangibles | [1-5] |  | 4.1 ± 0.9 | 0.83 | [0.79, 0.86] | <0.001*** |
| pReliability | | | | | | |
| Education | College or above | 322 | 4.3 ± 0.9 |  | | |
|  | Middle school | 466 | 3.9 ± 0.9 | 0.05 | [−0.02, 0.13] | 0.177 |
|  | Primary school or below | 245 | 4.2 ± 0.7 | 0.14 | [0.05, 0.22] | 0.001** |
| Medical insurance | CI | 91 | 4.1 ± 1.0 |  | | |
|  | NCMS | 200 | 3.7 ± 0.7 | −0.10 | [−0.17, −0.02] | 0.011* |
|  | SMI | 84 | 4.5 ± 0.5 | 0.24 | [0.15, 0.33] | <0.001*** |
|  | URBMI | 424 | 3.8 ± 0.9 | −0.00 | [−0.11, 0.10] | 0.940 |
|  | UWM | 234 | 4.3 ± 0.4 | 0.04 | [−0.07, 0.15] | 0.435 |
| eReliability | [1-5] |  | 4.1 ± 0.9 | 0.79 | [0.76, 0.83] | <0.001 |
|  |  |  |  |  |  |  |
| pResponsiveness | | | | | | |
| Age | <40 | 504 | 4.0 ± 0.9 |  | | |
|  | 40-59 | 336 | 4.1 ± 0.9 | 0.04 | [−0.02, 0.11] | 0.210 |
|  | >60 | 189 | 4.2 ± 0.8 | 0.15 | [0.06, 0.24] | <0.001*** |
| Education | College or above | 322 | 4.3 ± 0.9 |  | | |
|  | Middle school | 466 | 3.8 ± 0.9 | 0.02 | [−0.06, 0.10] | 0.570 |
|  | Primary school or below | 245 | 4.2 ± 0.7 | 0.11 | [0.02, 0.20] | 0.019* |
| Medical insurance | CI | 91 | 4.1 ± 1.0 |  | | |
|  | NCMS | 200 | 3.6 ± 0.7 | −0.10 | [−0.17, −0.03] | 0.008** |
|  | SMI | 84 | 4.4 ± 0.7 | 0.15 | [0.06, 0.23] | 0.001** |
|  | URBMI | 424 | 3.8 ± 0.9 | 0.02 | [−0.08, 0.12] | 0.710 |
|  | UWM | 234 | 4.3 ± 0.5 | 0.02 | [−0.09, 0.14] | 0.657 |
| Household register | Rural | 562 | 4.0 ± 0.9 |  | | |
|  | Urban | 471 | 4.0 ± 0.9 | −0.06 | [−0.12, −0.00] | 0.034* |
| Department | Genera | 632 | 4.3 ± 0.8 |  | | |
|  | Internal | 195 | 4.0 ± 0.7 | 0.10 | [0.01, 0.20] | 0.034* |
|  | Surgery | 206 | 4.0 ± 0.9 | 0.10 | [0.02, 0.18] | 0.012* |
| Visits | 1-5 | 560 | 4.1 ± 0.8 |  | | |
|  | 11-15 | 175 | 4.1 ± 0.8 | −0.01 | [−0.08, 0.06] | 0.772 |
|  | 16+ | 88 | 4.0 ± 0.9 | −0.08 | [−0.16, −0.00] | 0.046* |
|  | 6-10 | 210 | 3.4 ± 1.0 | −0.17 | [−0.28, −0.07] | 0.001** |
| eResponsiveness | [1-5] |  | 4.0 ± 0.9 | 0.85 | [0.82, 0.89] | <0.001*** |
|  |  |  |  |  |  |  |
| pAssurance | | | | | | |
| Education | College or above | 322 | 4.3 ± 0.9 |  | | |
|  | Middle school | 466 | 3.8 ± 1.0 | −0.08 | [−0.17, 0.01] | 0.070 |
|  | Primary school or below | 245 | 4.2 ± 0.7 | 0.04 | [−0.06, 0.14] | 0.450 |
| Medical insurance | CI | 91 | 4.1 ± 1.0 |  | | |
|  | NCMS | 200 | 3.6 ± 0.8 | −0.10 | [−0.19, −0.02] | 0.020* |
|  | SMI | 84 | 4.5 ± 0.6 | 0.18 | [0.08, 0.28] | <0.001*** |
|  | URBMI | 424 | 3.7 ± 0.9 | −0.10 | [−0.22, 0.02] | 0.106 |
|  | UWM | 234 | 4.3 ± 0.5 | 0.03 | [−0.10, 0.15] | 0.683 |
| Hospital | Y1 | 522 | 3.9 ± 1.0 |  | | |
|  | Y2 | 79 | 4.0 ± 0.7 | 0.00 | [−0.13, 0.13] | 0.981 |
|  | Y3 | 132 | 4.5 ± 0.8 | 0.16 | [0.06, 0.27] | 0.002** |
|  | Y4 | 120 | 4.1 ± 0.8 | 0.06 | [−0.05, 0.16] | 0.300 |
|  | Y5 | 92 | 4.0 ± 0.9 | −0.02 | [−0.14, 0.10] | 0.718 |
|  | Y6 | 88 | 4.1 ± 0.8 | 0.05 | [−0.07, 0.17] | 0.445 |
| eAssurance | [1-5] |  | 4.1 ± 0.9 | 0.75 | [0.71, 0.78] | <0.001*** |
| pEmpathy | | | | | | |
| Sex | Female | 543 | 4.2 ± 0.8 |  | | |
|  | Male | 490 | 4.1 ± 0.9 | −0.07 | [−0.13, −0.01] | 0.030* |
| Education | College or above | 322 | 4.4 ± 0.9 |  | | |
|  | Middle school | 466 | 3.9 ± 0.9 | −0.09 | [−0.18, −0.01] | 0.034* |
|  | Primary school or below | 245 | 4.3 ± 0.7 | −0.05 | [−0.15, 0.06] | 0.361 |
| Job | Admin. Staff | 32 | 4.3 ± 1.0 |  | | |
|  | Bus. & Serv. Staff | 229 | 4.0 ± 1.1 | 0.04 | [−0.11, 0.18] | 0.617 |
|  | Ent. Staff | 110 | 4.1 ± 0.8 | 0.17 | [0.04, 0.29] | 0.009** |
|  | Famer | 102 | 4.3 ± 0.7 | 0.03 | [−0.18, 0.24] | 0.804 |
|  | P&T Equip. Ops. & Assoc. Staff | 105 | 3.9 ± 0.8 | 0.09 | [−0.05, 0.24] | 0.212 |
|  | Prof. Tech. Staff | 89 | 4.4 ± 0.6 | 0.18 | [0.02, 0.34] | 0.028* |
|  | Retire | 104 | 3.1 ± 0.9 | −0.17 | [−0.50, 0.15] | 0.297 |
|  | Soldier | 10 | 4.3 ± 0.5 | 0.24 | [0.10, 0.39] | 0.001** |
|  | Student | 172 | 3.7 ± 0.9 | −0.01 | [−0.15, 0.13] | 0.860 |
|  | Unemp. | 80 | 4.3 ± 0.8 | 0.13 | [−0.02, 0.27] | 0.093 |
| Medical insurance | CI | 91 | 4.1 ± 1.0 |  | | |
|  | NCMS | 200 | 3.7 ± 0.8 | −0.08 | [−0.16, 0.01] | 0.069 |
|  | SMI | 84 | 4.6 ± 0.6 | 0.17 | [0.07, 0.27] | <0.001*** |
|  | URBMI | 424 | 3.8 ± 0.8 | −0.08 | [−0.09, 0.04] | 0.188 |
|  | UWM | 234 | 4.3 ± 0.5 | −0.09 | [−0.25, 0.06] | 0.248 |
| Department | Genera | 632 | 4.4 ± 0.8 |  | | |
|  | Internal | 195 | 4.0 ± 0.8 | 0.03 | [−0.07, 0.14] | 0.536 |
|  | Surgery | 206 | 4.1 ± 0.9 | 0.11 | [0.02, 0.19] | 0.014* |
| Visits | 1-5 | 560 | 4.2 ± 0.8 |  | | |
|  | 11-15 | 175 | 4.2 ± 0.7 | 0.03 | [−0.05, 0.11] | 0.489 |
|  | 16+ | 88 | 4.1 ± 0.9 | 0.00 | [−0.08, 0.09] | 0.976 |
|  | 6-10 | 210 | 3.5 ± 1.1 | −0.19 | [−0.31, −0.07] | 0.001** |
| eEmpathy | [1-5] |  | 4.1 ± 0.9 | 0.74 | [0.70, 0.78] | <0.001*** |

**Notes:**

**Abbreviations:** *P < 0.05, **P < 0.01, ***P < 0.001. CI: Condence interval

**Supplementary Table 9** Exploratory Structural Equation Modeling Analysis

| project | item | question | Scale Mean if Item Deleted | Scale Variance if Item Deleted | Corrected Item-Total Correlation | Squared Multiple Correlation | Cronbach's Alpha if Item Deleted | Cronbach's Alpha | N of Items |
| --- | --- | --- | --- | --- | --- | --- | --- | --- | --- |
| Expectation | Tangibles | Tangibles1 | 16.30 | 11.976 | .739 | .548 | .862 | .889 | 5 |
|  |  | Tangibles2 | 16.26 | 12.022 | .710 | .510 | .869 |  |  |
|  |  | Tangibles3 | 16.23 | 12.403 | .702 | .500 | .871 |  |  |
|  |  | Tangibles4 | 16.29 | 11.867 | .744 | .558 | .861 |  |  |
|  |  | Tangibles5 | 16.32 | 11.702 | .751 | .566 | .859 |  |  |
|  | Reliability | Reliability1 | 16.17 | 12.626 | .740 | .548 | .877 | .898 | 5 |
|  |  | Reliability2 | 16.25 | 12.110 | .745 | .562 | .876 |  |  |
|  |  | Reliability3 | 16.20 | 12.371 | .749 | .563 | .875 |  |  |
|  |  | Reliability4 | 16.28 | 11.771 | .770 | .594 | .870 |  |  |
|  |  | Reliability5 | 16.28 | 12.148 | .736 | .547 | .878 |  |  |
|  | Responsiveness | Responsiveness1 | 16.21 | 11.826 | .758 | .579 | .863 | .892 | 5 |
|  |  | Responsiveness2 | 16.18 | 12.021 | .756 | .586 | .864 |  |  |
|  |  | Responsiveness3 | 16.20 | 11.926 | .748 | .571 | .866 |  |  |
|  |  | Responsiveness4 | 16.18 | 12.154 | .696 | .502 | .877 |  |  |
|  |  | Responsiveness5 | 16.19 | 12.334 | .720 | .543 | .872 |  |  |
|  | Assurance | Assurance1 | 12.16 | 7.874 | .738 | .545 | .866 | .890 | 4 |
|  |  | Assurance2 | 12.18 | 7.747 | .784 | .620 | .849 |  |  |
|  |  | Assurance3 | 12.25 | 7.598 | .751 | .564 | .862 |  |  |
|  |  | Assurance4 | 12.14 | 7.910 | .762 | .589 | .857 |  |  |
|  | Empathy | Empathy1 | 12.31 | 6.801 | .741 | .549 | .833 | .873 | 4 |
|  |  | Empathy2 | 12.29 | 7.044 | .733 | .543 | .836 |  |  |
|  |  | Empathy3 | 12.28 | 7.126 | .739 | .548 | .834 |  |  |
|  |  | Empathy4 | 12.31 | 6.940 | .703 | .496 | .848 |  |  |
| Perception | Tangibles | Tangibles1 | 16.27 | 12.383 | .751 | .568 | .880 | .901 | 5 |
|  |  | Tangibles2 | 16.25 | 12.369 | .732 | .537 | .884 |  |  |
|  |  | Tangibles3 | 16.27 | 12.169 | .780 | .610 | .874 |  |  |
|  |  | Tangibles4 | 16.27 | 12.448 | .760 | .581 | .878 |  |  |
|  |  | Tangibles5 | 16.26 | 12.347 | .746 | .561 | .881 |  |  |
|  | Reliability | Reliability1 | 16.26 | 12.302 | .714 | .512 | .885 | .899 | 5 |
|  |  | Reliability2 | 16.27 | 12.008 | .747 | .561 | .878 |  |  |
|  |  | Reliability3 | 16.29 | 11.737 | .784 | .620 | .870 |  |  |
|  |  | Reliability4 | 16.27 | 11.861 | .751 | .572 | .877 |  |  |
|  |  | Reliability5 | 16.33 | 11.607 | .754 | .569 | .876 |  |  |
|  | Responsiveness | Responsiveness1 | 16.12 | 13.035 | .713 | .511 | .886 | .900 | 5 |
|  |  | Responsiveness2 | 16.10 | 13.071 | .748 | .570 | .878 |  |  |
|  |  | Responsiveness3 | 16.18 | 12.628 | .746 | .567 | .879 |  |  |
|  |  | Responsiveness4 | 16.11 | 12.620 | .772 | .604 | .873 |  |  |
|  |  | Responsiveness5 | 16.16 | 12.541 | .775 | .603 | .872 |  |  |
|  | Assurance | Assurance1 | 12.14 | 7.793 | .731 | .539 | .853 | .882 | 4 |
|  |  | Assurance2 | 12.14 | 7.645 | .746 | .565 | .847 |  |  |
|  |  | Assurance3 | 12.14 | 7.674 | .740 | .552 | .850 |  |  |
|  |  | Assurance4 | 12.12 | 7.623 | .755 | .577 | .844 |  |  |
|  | Empathy | Empathy1 | 12.35 | 6.868 | .713 | .518 | .829 | .866 | 4 |
|  |  | Empathy2 | 12.32 | 6.959 | .712 | .513 | .830 |  |  |
|  |  | Empathy3 | 12.34 | 6.725 | .742 | .560 | .817 |  |  |
|  |  | Empathy4 | 12.38 | 6.680 | .696 | .486 | .837 |  |  |

**Supplementary Table 10** Confirmatory Analysis of the Structural Equation Model

| project | item | question | | | β | S.E. | C.R. | P | CR | AVE |
| --- | --- | --- | --- | --- | --- | --- | --- | --- | --- | --- |
| Expectation | Assurance | b19 | <--- | Assurance | 0.816 | \ | \ | \ | 0.8904 | 0.67 |
|  |  | b18 | <--- | Assurance | 0.831 | 0.034 | 32.009 | *** |  |  |
|  |  | b17 | <--- | Assurance | 0.819 | 0.032 | 31.282 | *** |  |  |
|  |  | b16 | <--- | Assurance | 0.808 | 0.033 | 30.655 | *** |  |  |
|  | Empathy | b23 | <--- | Empathy | 0.787 | \ | \ | \ | 0.8734 | 0.6331 |
|  |  | b22 | <--- | Empathy | 0.823 | 0.033 | 29.771 | *** |  |  |
|  |  | b21 | <--- | Empathy | 0.781 | 0.034 | 27.802 | *** |  |  |
|  |  | b20 | <--- | Empathy | 0.791 | 0.035 | 28.28 | *** |  |  |
|  | Tangibles | b5 | <--- | Tangibles | 0.82 | \ | \ | \ | 0.8883 | 0.6143 |
|  |  | b4 | <--- | Tangibles | 0.799 | 0.031 | 30.374 | *** |  |  |
|  |  | b3 | <--- | Tangibles | 0.757 | 0.031 | 28.115 | *** |  |  |
|  |  | b2 | <--- | Tangibles | 0.777 | 0.032 | 29.178 | *** |  |  |
|  |  | b1 | <--- | Tangibles | 0.764 | 0.032 | 28.503 | *** |  |  |
|  | Reliability | b10 | <--- | Reliability | 0.799 | \ | \ | \ | 0.898 | 0.6378 |
|  |  | b9 | <--- | Reliability | 0.809 | 0.035 | 29.948 | *** |  |  |
|  |  | b8 | <--- | Reliability | 0.8 | 0.032 | 29.508 | *** |  |  |
|  |  | b7 | <--- | Reliability | 0.795 | 0.034 | 29.254 | *** |  |  |
|  |  | b6 | <--- | Reliability | 0.791 | 0.031 | 29.041 | *** |  |  |
|  | Responsiveness | b15 | <--- | Responsiveness | 0.77 | \ | \ | \ | 0.8924 | 0.624 |
|  |  | b14 | <--- | Responsiveness | 0.775 | 0.039 | 27.149 | *** |  |  |
|  |  | b13 | <--- | Responsiveness | 0.8 | 0.038 | 28.219 | *** |  |  |
|  |  | b12 | <--- | Responsiveness | 0.799 | 0.037 | 28.197 | *** |  |  |
|  |  | b11 | <--- | Responsiveness | 0.805 | 0.039 | 28.442 | *** |  |  |
| Perception | Assurance | b42 | <--- | Assurance | 0.823 | \ | \ | \ | 0.8813 | 0.6501 |
|  |  | b41 | <--- | Assurance | 0.817 | 0.031 | 31.764 | *** |  |  |
|  |  | b40 | <--- | Assurance | 0.813 | 0.031 | 31.55 | *** |  |  |
|  |  | b39 | <--- | Assurance | 0.771 | 0.032 | 29.168 | *** |  |  |
|  | Empathy | b46 | <--- | Empathy | 0.773 | \ | \ | \ | 0.8665 | 0.6187 |
|  |  | b45 | <--- | Empathy | 0.8 | 0.035 | 28.278 | *** |  |  |
|  |  | b44 | <--- | Empathy | 0.78 | 0.034 | 27.39 | *** |  |  |
|  |  | b43 | <--- | Empathy | 0.793 | 0.035 | 27.958 | *** |  |  |
|  | Tangibles | b28 | <--- | Tangibles | 0.802 | \ | \ | \ | 0.9015 | 0.6469 |
|  |  | b27 | <--- | Tangibles | 0.815 | 0.033 | 30.205 | *** |  |  |
|  |  | b26 | <--- | Tangibles | 0.819 | 0.033 | 30.414 | *** |  |  |
|  |  | b25 | <--- | Tangibles | 0.794 | 0.034 | 29.133 | *** |  |  |
|  |  | b24 | <--- | Tangibles | 0.791 | 0.034 | 28.965 | *** |  |  |
|  | Reliability | b33 | <--- | Reliability | 0.801 | \ | \ | \ | 0.8996 | 0.6419 |
|  |  | b32 | <--- | Reliability | 0.808 | 0.032 | 30.121 | *** |  |  |
|  |  | b31 | <--- | Reliability | 0.815 | 0.032 | 30.53 | *** |  |  |
|  |  | b30 | <--- | Reliability | 0.813 | 0.031 | 30.413 | *** |  |  |
|  |  | b29 | <--- | Reliability | 0.768 | 0.032 | 28.125 | *** |  |  |
|  | Responsiveness | b38 | <--- | Responsiveness | 0.809 | \ | \ | \ | 0.9001 | 0.6432 |
|  |  | b37 | <--- | Responsiveness | 0.827 | 0.032 | 31.61 | *** |  |  |
|  |  | b36 | <--- | Responsiveness | 0.79 | 0.033 | 29.594 | *** |  |  |
|  |  | b35 | <--- | Responsiveness | 0.805 | 0.031 | 30.412 | *** |  |  |
|  |  | b34 | <--- | Responsiveness | 0.778 | 0.033 | 28.993 | *** |  |  |

**Notes:**

**Abbreviations:** *P < 0.05, **P < 0.01, ***P < 0.001.

**Supplementary Table 11** Model Fit Indices

| Summary of Overall Model Fit Indices | | | | |
| --- | --- | --- | --- | --- |
| Fit Statistic | Criterion (Threshold) | Initial Model | Modified Model | Meets Criterion |
| Absolute Fit Measures | | | | |
| χ² | P > 0.05 | 462.041 | 368.41 | No |
| χ²/df | < 2 (Good), < 3 (Acceptable) | 7.452 | 6.244 | No |
| RMR | < 0.05 | 0.01 | 0.008 | Yes |
| RMSEA | < 0.05 (Good), < 0.08 (Acceptable) | 0.079 | 0.071 | Yes |
| SRMR | < 0.05 (Good), < 0.08 (Acceptable) | 0.0285 | 0.0154 | Yes |
| GFI | >0.9 | 0.937 | 0.949 | Yes |
| AGFI | >0.9 | 0.908 | 0.922 | Yes |
| CN | >200 | 182 | 219 | Yes |
| Comparative/Incremental Fit Measures | | | | |
| NFI | > 0.9 (Acceptable), ≥ 0.95 (Good) | 0.972 | 0.978 | Yes |
| RFI | > 0.9 (Acceptable), ≥ 0.95 (Good) | 0.965 | 0.971 | Yes |
| IFI | > 0.9 (Acceptable), ≥ 0.95 (Good) | 0.976 | 0.981 | Yes |
| TLI(NNFI) | > 0.9 (Acceptable), ≥ 0.95 (Good) | 0.97 | 0.975 | Yes |
| CFI | > 0.9 (Acceptable), ≥ 0.95 (Good) | 0.976 | 0.981 | Yes |
| Parsimony-Adjusted Fit Measures | | | | |
| PGFI | >0.5 | 0.638 | 0.616 | Yes |
| PNFI | >0.5 | 0.773 | 0.74 | Yes |
| PCFI | >0.5 | 0.776 | 0.742 | Yes |
