# Supplementary material for: A cross-sectional study on patient-centered outpatient service quality in the county-level integrated health organization of Yuhuan, Zhejiang province of China
Source: Front Public Health. 2026 Sep 23;14:1889257. doi: 10.3389/fpubh.2026.1889257 (PMC13642747; doi:10.3389/fpubh.2026.1889257)
Supplement: Supplementary file 2 [file Supplementary_file_2.docx]

#### C:\Users\Lenovo\Desktop\新建文件夹 (4)\初始模型.amw

##### Analysis Summary

##### Date and Time

Date: 2025年9月23日

Time: 16:12:00

##### Title

初始模型: 2025年9月23日 16:12

##### Groups

##### Group number 1 (Group number 1)

##### Notes for Group (Group number 1)

The model is recursive.

Sample size = 1033

##### Variable Summary (Group number 1)

##### Your model contains the following variables (Group number 1)

Observed, endogenous variables

Tangibles_P

Reliability_P

Responsiveness_P

Assurance_P

Empathy_P

c2

c3

Empathy_E

Assurance_E

Responsiveness_E

Reliability_E

Tangibles_E

c1

Unobserved, endogenous variables

Perception

BI

Unobserved, exogenous variables

e1

e2

e3

e4

e5

e6

e7

Expectation

e8

e9

e10

e11

e12

e13

e14

e15

##### Variable counts (Group number 1)

| Number of variables in your model: | 31 |
| --- | --- |
| Number of observed variables: | 13 |
| Number of unobserved variables: | 18 |
| Number of exogenous variables: | 16 |
| Number of endogenous variables: | 15 |

##### Parameter Summary (Group number 1)

|  | Weights | Covariances | Variances | Means | Intercepts | Total |
| --- | --- | --- | --- | --- | --- | --- |
| Fixed | 18 | 0 | 0 | 0 | 0 | 18 |
| Labeled | 0 | 0 | 0 | 0 | 0 | 0 |
| Unlabeled | 13 | 0 | 16 | 0 | 0 | 29 |
| Total | 31 | 0 | 16 | 0 | 0 | 47 |

##### Assessment of normality (Group number 1)

| Variable | min | max | skew | c.r. | kurtosis | c.r. |
| --- | --- | --- | --- | --- | --- | --- |
| c1 | 1.000 | 5.000 | -.881 | -11.563 | .262 | 1.719 |
| Tangibles_E | 1.000 | 5.000 | -.871 | -11.431 | .200 | 1.311 |
| Reliability_E | 1.000 | 5.000 | -.881 | -11.553 | .261 | 1.711 |
| Responsiveness_E | 1.000 | 5.000 | -.823 | -10.798 | .103 | .678 |
| Assurance_E | 1.000 | 5.000 | -.996 | -13.067 | .481 | 3.158 |
| Empathy_E | 1.000 | 5.000 | -.864 | -11.332 | .230 | 1.506 |
| c3 | .000 | 1.000 | -2.585 | -33.917 | 4.682 | 30.714 |
| c2 | .000 | 1.000 | -4.416 | -57.946 | 17.503 | 114.831 |
| Empathy_P | 1.250 | 5.000 | -.812 | -10.650 | .038 | .252 |
| Assurance_P | 1.000 | 5.000 | -.865 | -11.347 | .116 | .760 |
| Responsiveness_P | 1.200 | 5.000 | -.837 | -10.977 | .140 | .919 |
| Reliability_P | 1.000 | 5.000 | -.828 | -10.859 | .143 | .940 |
| Tangibles_P | 1.200 | 5.000 | -.909 | -11.925 | .308 | 2.021 |
| Multivariate |  |  |  |  | 184.898 | 150.460 |

##### Observations farthest from the centroid (Mahalanobis distance) (Group number 1)

| Observation number | Mahalanobis d-squared | p1 | p2 |
| --- | --- | --- | --- |
| 669 | 166.595 | .000 | .000 |
| 789 | 87.154 | .000 | .000 |
| 1019 | 84.350 | .000 | .000 |
| 932 | 78.612 | .000 | .000 |
| 818 | 76.625 | .000 | .000 |
| 1017 | 74.924 | .000 | .000 |
| 1015 | 73.979 | .000 | .000 |
| 923 | 72.009 | .000 | .000 |
| 995 | 67.138 | .000 | .000 |
| 787 | 67.054 | .000 | .000 |
| 796 | 66.373 | .000 | .000 |
| 1011 | 65.928 | .000 | .000 |
| 1031 | 64.755 | .000 | .000 |
| 1022 | 62.094 | .000 | .000 |
| 1024 | 61.820 | .000 | .000 |
| 822 | 61.239 | .000 | .000 |
| 1029 | 59.433 | .000 | .000 |
| 874 | 57.410 | .000 | .000 |
| 831 | 56.603 | .000 | .000 |
| 1006 | 55.855 | .000 | .000 |
| 1027 | 54.134 | .000 | .000 |
| 914 | 54.075 | .000 | .000 |
| 842 | 53.720 | .000 | .000 |
| 1013 | 53.072 | .000 | .000 |
| 1028 | 52.972 | .000 | .000 |
| 1021 | 52.746 | .000 | .000 |
| 913 | 52.566 | .000 | .000 |
| 1018 | 51.141 | .000 | .000 |
| 880 | 51.053 | .000 | .000 |
| 912 | 51.009 | .000 | .000 |
| 896 | 50.890 | .000 | .000 |
| 1016 | 50.189 | .000 | .000 |
| 828 | 49.434 | .000 | .000 |
| 798 | 48.943 | .000 | .000 |
| 56 | 48.927 | .000 | .000 |
| 715 | 48.415 | .000 | .000 |
| 1012 | 48.156 | .000 | .000 |
| 994 | 47.895 | .000 | .000 |
| 742 | 47.753 | .000 | .000 |
| 809 | 47.523 | .000 | .000 |
| 991 | 47.417 | .000 | .000 |
| 1004 | 46.689 | .000 | .000 |
| 11 | 46.600 | .000 | .000 |
| 847 | 46.306 | .000 | .000 |
| 1000 | 46.147 | .000 | .000 |
| 978 | 46.020 | .000 | .000 |
| 841 | 45.218 | .000 | .000 |
| 762 | 44.931 | .000 | .000 |
| 732 | 44.225 | .000 | .000 |
| 817 | 43.810 | .000 | .000 |
| 1002 | 43.578 | .000 | .000 |
| 34 | 43.446 | .000 | .000 |
| 8 | 42.459 | .000 | .000 |
| 5 | 41.383 | .000 | .000 |
| 920 | 40.692 | .000 | .000 |
| 829 | 40.444 | .000 | .000 |
| 998 | 39.757 | .000 | .000 |
| 834 | 39.702 | .000 | .000 |
| 1 | 39.537 | .000 | .000 |
| 996 | 39.335 | .000 | .000 |
| 933 | 39.257 | .000 | .000 |
| 371 | 39.226 | .000 | .000 |
| 791 | 39.173 | .000 | .000 |
| 39 | 39.062 | .000 | .000 |
| 879 | 38.767 | .000 | .000 |
| 917 | 38.352 | .000 | .000 |
| 1007 | 38.024 | .000 | .000 |
| 3 | 37.502 | .000 | .000 |
| 488 | 37.454 | .000 | .000 |
| 581 | 37.111 | .000 | .000 |
| 10 | 36.866 | .000 | .000 |
| 827 | 36.842 | .000 | .000 |
| 882 | 36.778 | .000 | .000 |
| 713 | 36.691 | .000 | .000 |
| 180 | 36.672 | .000 | .000 |
| 800 | 36.624 | .000 | .000 |
| 1032 | 36.443 | .001 | .000 |
| 2 | 36.021 | .001 | .000 |
| 790 | 35.977 | .001 | .000 |
| 6 | 35.964 | .001 | .000 |
| 918 | 35.930 | .001 | .000 |
| 1003 | 34.950 | .001 | .000 |
| 766 | 34.906 | .001 | .000 |
| 988 | 34.855 | .001 | .000 |
| 889 | 34.635 | .001 | .000 |
| 924 | 34.393 | .001 | .000 |
| 4 | 34.102 | .001 | .000 |
| 812 | 34.003 | .001 | .000 |
| 370 | 33.961 | .001 | .000 |
| 9 | 33.701 | .001 | .000 |
| 722 | 33.296 | .002 | .000 |
| 921 | 32.770 | .002 | .000 |
| 45 | 32.769 | .002 | .000 |
| 665 | 32.259 | .002 | .000 |
| 890 | 32.238 | .002 | .000 |
| 916 | 32.033 | .002 | .000 |
| 938 | 31.992 | .002 | .000 |
| 342 | 31.897 | .002 | .000 |
| 909 | 31.746 | .003 | .000 |
| 786 | 31.644 | .003 | .000 |

##### Models

##### Default model (Default model)

##### Notes for Model (Default model)

##### Computation of degrees of freedom (Default model)

| Number of distinct sample moments: | 91 |
| --- | --- |
| Number of distinct parameters to be estimated: | 29 |
| Degrees of freedom (91 - 29): | 62 |

##### Result (Default model)

Minimum was achieved

Chi-square = 462.041

Degrees of freedom = 62

Probability level = .000

##### Group number 1 (Group number 1 - Default model)

##### Estimates (Group number 1 - Default model)

##### Scalar Estimates (Group number 1 - Default model)

##### Maximum Likelihood Estimates

##### Regression Weights: (Group number 1 - Default model)

|  |  |  | Estimate | S.E. | C.R. | P | Label |
| --- | --- | --- | --- | --- | --- | --- | --- |
| Perception | <--- | Expectation | .971 | .021 | 46.046 | *** |  |
| c1 | <--- | Perception | .908 | .114 | 7.940 | *** |  |
| c1 | <--- | Expectation | .007 | .116 | .065 | .948 |  |
| BI | <--- | c1 | .051 | .006 | 8.041 | *** |  |
| Tangibles_P | <--- | Perception | 1.000 |  |  |  |  |
| Reliability_P | <--- | Perception | .999 | .017 | 57.271 | *** |  |
| Responsiveness_P | <--- | Perception | 1.038 | .018 | 57.845 | *** |  |
| Assurance_P | <--- | Perception | 1.042 | .019 | 53.465 | *** |  |
| Empathy_P | <--- | Perception | .971 | .019 | 52.203 | *** |  |
| c2 | <--- | BI | 1.000 |  |  |  |  |
| c3 | <--- | BI | 1.191 | .195 | 6.116 | *** |  |
| Empathy_E | <--- | Expectation | 1.000 |  |  |  |  |
| Assurance_E | <--- | Expectation | 1.071 | .021 | 51.935 | *** |  |
| Responsiveness_E | <--- | Expectation | 1.021 | .019 | 54.809 | *** |  |
| Reliability_E | <--- | Expectation | 1.022 | .019 | 53.773 | *** |  |
| Tangibles_E | <--- | Expectation | .996 | .019 | 51.073 | *** |  |

##### Standardized Regression Weights: (Group number 1 - Default model)

|  |  |  | Estimate |
| --- | --- | --- | --- |
| Perception | <--- | Expectation | .955 |
| c1 | <--- | Perception | .741 |
| c1 | <--- | Expectation | .006 |
| BI | <--- | c1 | .357 |
| Tangibles_P | <--- | Perception | .924 |
| Reliability_P | <--- | Perception | .940 |
| Responsiveness_P | <--- | Perception | .943 |
| Assurance_P | <--- | Perception | .922 |
| Empathy_P | <--- | Perception | .915 |
| c2 | <--- | BI | .680 |
| c3 | <--- | BI | .546 |
| Empathy_E | <--- | Expectation | .914 |
| Assurance_E | <--- | Expectation | .924 |
| Responsiveness_E | <--- | Expectation | .939 |
| Reliability_E | <--- | Expectation | .934 |
| Tangibles_E | <--- | Expectation | .919 |

##### Variances: (Group number 1 - Default model)

|  |  |  | Estimate | S.E. | C.R. | P | Label |
| --- | --- | --- | --- | --- | --- | --- | --- |
| Expectation |  |  | .621 | .032 | 19.143 | *** |  |
| e13 |  |  | .057 | .004 | 12.678 | *** |  |
| e15 |  |  | .426 | .019 | 21.867 | *** |  |
| e14 |  |  | .017 | .003 | 5.265 | *** |  |
| e1 |  |  | .110 | .006 | 19.440 | *** |  |
| e2 |  |  | .084 | .005 | 18.416 | *** |  |
| e3 |  |  | .087 | .005 | 18.204 | *** |  |
| e4 |  |  | .123 | .006 | 19.536 | *** |  |
| e5 |  |  | .118 | .006 | 19.823 | *** |  |
| e6 |  |  | .023 | .003 | 6.881 | *** |  |
| e7 |  |  | .066 | .005 | 12.293 | *** |  |
| e8 |  |  | .123 | .006 | 19.674 | *** |  |
| e9 |  |  | .122 | .006 | 19.209 | *** |  |
| e10 |  |  | .086 | .005 | 18.185 | *** |  |
| e11 |  |  | .095 | .005 | 18.596 | *** |  |
| e12 |  |  | .113 | .006 | 19.454 | *** |  |

##### Squared Multiple Correlations: (Group number 1 - Default model)

|  |  |  | Estimate |
| --- | --- | --- | --- |
| Perception |  |  | .912 |
| c1 |  |  | .558 |
| BI |  |  | .128 |
| Tangibles_E |  |  | .844 |
| Reliability_E |  |  | .872 |
| Responsiveness_E |  |  | .882 |
| Assurance_E |  |  | .853 |
| Empathy_E |  |  | .835 |
| c3 |  |  | .298 |
| c2 |  |  | .462 |
| Empathy_P |  |  | .837 |
| Assurance_P |  |  | .850 |
| Responsiveness_P |  |  | .888 |
| Reliability_P |  |  | .884 |
| Tangibles_P |  |  | .853 |

##### Matrices (Group number 1 - Default model)

##### Factor Score Weights (Group number 1 - Default model)

|  | c1 | Tangibles_E | Reliability_E | Responsiveness_E | Assurance_E | Empathy_E | c3 | c2 | Empathy_P | Assurance_P | Responsiveness_P | Reliability_P | Tangibles_P |
| --- | --- | --- | --- | --- | --- | --- | --- | --- | --- | --- | --- | --- | --- |
| Expectation | .009 | .138 | .170 | .187 | .138 | .129 | .000 | .000 | .032 | .033 | .046 | .046 | .035 |
| Perception | .033 | .034 | .042 | .046 | .034 | .032 | .000 | .000 | .126 | .129 | .183 | .181 | .139 |
| BI | .024 | .000 | .000 | .000 | .000 | .000 | .147 | .354 | .000 | .000 | .000 | .000 | .000 |

##### Total Effects (Group number 1 - Default model)

|  | Expectation | Perception | c1 | BI |
| --- | --- | --- | --- | --- |
| Perception | .971 | .000 | .000 | .000 |
| c1 | .889 | .908 | .000 | .000 |
| BI | .045 | .046 | .051 | .000 |
| Tangibles_E | .996 | .000 | .000 | .000 |
| Reliability_E | 1.022 | .000 | .000 | .000 |
| Responsiveness_E | 1.021 | .000 | .000 | .000 |
| Assurance_E | 1.071 | .000 | .000 | .000 |
| Empathy_E | 1.000 | .000 | .000 | .000 |
| c3 | .054 | .055 | .061 | 1.191 |
| c2 | .045 | .046 | .051 | 1.000 |
| Empathy_P | .943 | .971 | .000 | .000 |
| Assurance_P | 1.012 | 1.042 | .000 | .000 |
| Responsiveness_P | 1.008 | 1.038 | .000 | .000 |
| Reliability_P | .970 | .999 | .000 | .000 |
| Tangibles_P | .971 | 1.000 | .000 | .000 |

##### Standardized Total Effects (Group number 1 - Default model)

|  | Expectation | Perception | c1 | BI |
| --- | --- | --- | --- | --- |
| Perception | .955 | .000 | .000 | .000 |
| c1 | .714 | .741 | .000 | .000 |
| BI | .255 | .265 | .357 | .000 |
| Tangibles_E | .919 | .000 | .000 | .000 |
| Reliability_E | .934 | .000 | .000 | .000 |
| Responsiveness_E | .939 | .000 | .000 | .000 |
| Assurance_E | .924 | .000 | .000 | .000 |
| Empathy_E | .914 | .000 | .000 | .000 |
| c3 | .139 | .144 | .195 | .546 |
| c2 | .173 | .180 | .243 | .680 |
| Empathy_P | .874 | .915 | .000 | .000 |
| Assurance_P | .880 | .922 | .000 | .000 |
| Responsiveness_P | .900 | .943 | .000 | .000 |
| Reliability_P | .898 | .940 | .000 | .000 |
| Tangibles_P | .882 | .924 | .000 | .000 |

##### Direct Effects (Group number 1 - Default model)

|  | Expectation | Perception | c1 | BI |
| --- | --- | --- | --- | --- |
| Perception | .971 | .000 | .000 | .000 |
| c1 | .007 | .908 | .000 | .000 |
| BI | .000 | .000 | .051 | .000 |
| Tangibles_E | .996 | .000 | .000 | .000 |
| Reliability_E | 1.022 | .000 | .000 | .000 |
| Responsiveness_E | 1.021 | .000 | .000 | .000 |
| Assurance_E | 1.071 | .000 | .000 | .000 |
| Empathy_E | 1.000 | .000 | .000 | .000 |
| c3 | .000 | .000 | .000 | 1.191 |
| c2 | .000 | .000 | .000 | 1.000 |
| Empathy_P | .000 | .971 | .000 | .000 |
| Assurance_P | .000 | 1.042 | .000 | .000 |
| Responsiveness_P | .000 | 1.038 | .000 | .000 |
| Reliability_P | .000 | .999 | .000 | .000 |
| Tangibles_P | .000 | 1.000 | .000 | .000 |

##### Standardized Direct Effects (Group number 1 - Default model)

|  | Expectation | Perception | c1 | BI |
| --- | --- | --- | --- | --- |
| Perception | .955 | .000 | .000 | .000 |
| c1 | .006 | .741 | .000 | .000 |
| BI | .000 | .000 | .357 | .000 |
| Tangibles_E | .919 | .000 | .000 | .000 |
| Reliability_E | .934 | .000 | .000 | .000 |
| Responsiveness_E | .939 | .000 | .000 | .000 |
| Assurance_E | .924 | .000 | .000 | .000 |
| Empathy_E | .914 | .000 | .000 | .000 |
| c3 | .000 | .000 | .000 | .546 |
| c2 | .000 | .000 | .000 | .680 |
| Empathy_P | .000 | .915 | .000 | .000 |
| Assurance_P | .000 | .922 | .000 | .000 |
| Responsiveness_P | .000 | .943 | .000 | .000 |
| Reliability_P | .000 | .940 | .000 | .000 |
| Tangibles_P | .000 | .924 | .000 | .000 |

##### Indirect Effects (Group number 1 - Default model)

|  | Expectation | Perception | c1 | BI |
| --- | --- | --- | --- | --- |
| Perception | .000 | .000 | .000 | .000 |
| c1 | .882 | .000 | .000 | .000 |
| BI | .045 | .046 | .000 | .000 |
| Tangibles_E | .000 | .000 | .000 | .000 |
| Reliability_E | .000 | .000 | .000 | .000 |
| Responsiveness_E | .000 | .000 | .000 | .000 |
| Assurance_E | .000 | .000 | .000 | .000 |
| Empathy_E | .000 | .000 | .000 | .000 |
| c3 | .054 | .055 | .061 | .000 |
| c2 | .045 | .046 | .051 | .000 |
| Empathy_P | .943 | .000 | .000 | .000 |
| Assurance_P | 1.012 | .000 | .000 | .000 |
| Responsiveness_P | 1.008 | .000 | .000 | .000 |
| Reliability_P | .970 | .000 | .000 | .000 |
| Tangibles_P | .971 | .000 | .000 | .000 |

##### Standardized Indirect Effects (Group number 1 - Default model)

|  | Expectation | Perception | c1 | BI |
| --- | --- | --- | --- | --- |
| Perception | .000 | .000 | .000 | .000 |
| c1 | .708 | .000 | .000 | .000 |
| BI | .255 | .265 | .000 | .000 |
| Tangibles_E | .000 | .000 | .000 | .000 |
| Reliability_E | .000 | .000 | .000 | .000 |
| Responsiveness_E | .000 | .000 | .000 | .000 |
| Assurance_E | .000 | .000 | .000 | .000 |
| Empathy_E | .000 | .000 | .000 | .000 |
| c3 | .139 | .144 | .195 | .000 |
| c2 | .173 | .180 | .243 | .000 |
| Empathy_P | .874 | .000 | .000 | .000 |
| Assurance_P | .880 | .000 | .000 | .000 |
| Responsiveness_P | .900 | .000 | .000 | .000 |
| Reliability_P | .898 | .000 | .000 | .000 |
| Tangibles_P | .882 | .000 | .000 | .000 |

##### Modification Indices (Group number 1 - Default model)

##### Covariances: (Group number 1 - Default model)

|  |  |  | M.I. | Par Change |
| --- | --- | --- | --- | --- |
| e14 | <--> | Expectation | 4.294 | .009 |
| e14 | <--> | e15 | 6.741 | -.010 |
| e11 | <--> | e12 | 9.508 | .012 |
| e10 | <--> | e15 | 7.056 | .018 |
| e10 | <--> | e11 | 4.772 | -.007 |
| e9 | <--> | e14 | 5.858 | .005 |
| e9 | <--> | e12 | 17.911 | -.018 |
| e9 | <--> | e10 | 7.441 | .010 |
| e8 | <--> | e15 | 13.374 | -.028 |
| e8 | <--> | e12 | 4.069 | .008 |
| e8 | <--> | e11 | 8.338 | -.011 |
| e8 | <--> | e10 | 4.424 | .008 |
| e7 | <--> | e12 | 10.698 | -.010 |
| e7 | <--> | e10 | 22.908 | .014 |
| e7 | <--> | e9 | 5.647 | .008 |
| e7 | <--> | e8 | 11.267 | -.011 |
| e6 | <--> | e12 | 11.008 | .007 |
| e6 | <--> | e10 | 17.149 | -.008 |
| e5 | <--> | e14 | 15.764 | -.008 |
| e5 | <--> | e12 | 46.623 | -.028 |
| e5 | <--> | e10 | 7.615 | -.010 |
| e5 | <--> | e9 | 10.469 | .014 |
| e5 | <--> | e8 | 9.089 | .013 |
| e5 | <--> | e6 | 12.721 | -.008 |
| e4 | <--> | e15 | 11.497 | -.026 |
| e4 | <--> | e14 | 5.121 | .005 |
| e4 | <--> | e9 | 9.179 | -.013 |
| e4 | <--> | e8 | 16.436 | .018 |
| e4 | <--> | e5 | 36.832 | .026 |
| e3 | <--> | e14 | 16.855 | .008 |
| e3 | <--> | e10 | 15.806 | .013 |
| e3 | <--> | e6 | 13.255 | .007 |
| e3 | <--> | e5 | 12.532 | -.013 |
| e3 | <--> | e4 | 4.490 | .008 |
| e2 | <--> | e15 | 13.084 | .024 |
| e2 | <--> | e12 | 4.512 | -.008 |
| e2 | <--> | e5 | 6.762 | .009 |
| e2 | <--> | e4 | 22.942 | -.018 |
| e1 | <--> | e13 | 5.250 | -.008 |
| e1 | <--> | e12 | 81.154 | .036 |
| e1 | <--> | e10 | 20.989 | -.017 |
| e1 | <--> | e5 | 4.097 | -.008 |

##### Variances: (Group number 1 - Default model)

|  |  |  | M.I. | Par Change |
| --- | --- | --- | --- | --- |

##### Regression Weights: (Group number 1 - Default model)

|  |  |  | M.I. | Par Change |
| --- | --- | --- | --- | --- |
| BI | <--- | Expectation | 4.294 | .015 |
| BI | <--- | Perception | 5.316 | .016 |
| Tangibles_E | <--- | c3 | 4.798 | -.081 |
| Tangibles_E | <--- | c2 | 5.425 | .128 |
| Tangibles_E | <--- | Empathy_P | 7.116 | -.035 |
| Tangibles_E | <--- | Tangibles_P | 9.735 | .041 |
| Responsiveness_E | <--- | c3 | 14.467 | .126 |
| Responsiveness_E | <--- | c2 | 5.028 | -.110 |
| Responsiveness_E | <--- | Tangibles_P | 4.016 | -.023 |
| Assurance_E | <--- | BI | 4.503 | .232 |
| Assurance_E | <--- | c3 | 7.277 | .104 |
| Empathy_E | <--- | c1 | 5.660 | -.028 |
| Empathy_E | <--- | BI | 6.099 | -.268 |
| Empathy_E | <--- | c3 | 12.882 | -.137 |
| c3 | <--- | Responsiveness_E | 6.660 | .026 |
| c2 | <--- | Tangibles_E | 5.388 | .016 |
| c2 | <--- | Responsiveness_P | 5.570 | .016 |
| Empathy_P | <--- | BI | 16.671 | -.433 |
| Empathy_P | <--- | Tangibles_E | 8.266 | -.038 |
| Empathy_P | <--- | c3 | 5.128 | -.084 |
| Empathy_P | <--- | c2 | 17.912 | -.234 |
| Empathy_P | <--- | Assurance_P | 4.901 | .028 |
| Assurance_P | <--- | c1 | 4.959 | -.027 |
| Assurance_P | <--- | Empathy_P | 5.375 | .032 |
| Responsiveness_P | <--- | BI | 12.592 | .336 |
| Responsiveness_P | <--- | c3 | 4.431 | .070 |
| Responsiveness_P | <--- | c2 | 15.977 | .197 |
| Reliability_P | <--- | c1 | 5.640 | .024 |
| Tangibles_P | <--- | Tangibles_E | 14.598 | .050 |

##### Minimization History (Default model)

| Iteration |  | Negative eigenvalues | Condition # | Smallest eigenvalue | Diameter | F | NTries | Ratio |
| --- | --- | --- | --- | --- | --- | --- | --- | --- |
| 0 | e | 6 |  | -1.324 | 9999.000 | 16225.015 | 0 | 9999.000 |
| 1 | e* | 13 |  | -.917 | 5.528 | 8434.959 | 20 | .131 |
| 2 | e* | 5 |  | -.207 | .629 | 5667.489 | 6 | .917 |
| 3 | e | 3 |  | -.312 | 1.470 | 3229.269 | 7 | .802 |
| 4 | e | 2 |  | -.536 | .989 | 1417.631 | 5 | 1.062 |
| 5 | e* | 1 |  | -.066 | .251 | 1014.990 | 6 | .762 |
| 6 | e | 0 | 1265.760 |  | .748 | 548.688 | 8 | .753 |
| 7 | e | 0 | 694.137 |  | .811 | 515.942 | 1 | .341 |
| 8 | e | 0 | 1014.262 |  | .145 | 468.314 | 1 | 1.164 |
| 9 | e | 0 | 1000.297 |  | .090 | 462.276 | 1 | 1.116 |
| 10 | e | 0 | 1017.819 |  | .020 | 462.041 | 1 | 1.030 |
| 11 | e | 0 | 1003.312 |  | .001 | 462.041 | 1 | 1.001 |

##### Model Fit Summary

##### CMIN

| Model | NPAR | CMIN | DF | P | CMIN/DF |
| --- | --- | --- | --- | --- | --- |
| Default model | 29 | 462.041 | 62 | .000 | 7.452 |
| Saturated model | 91 | .000 | 0 |  |  |
| Independence model | 13 | 16712.517 | 78 | .000 | 214.263 |

##### RMR, GFI

| Model | RMR | GFI | AGFI | PGFI |
| --- | --- | --- | --- | --- |
| Default model | .010 | .937 | .908 | .638 |
| Saturated model | .000 | 1.000 |  |  |
| Independence model | .487 | .148 | .007 | .127 |

##### Baseline Comparisons

| Model | NFI Delta1 | RFI rho1 | IFI Delta2 | TLI rho2 | CFI |
| --- | --- | --- | --- | --- | --- |
| Default model | .972 | .965 | .976 | .970 | .976 |
| Saturated model | 1.000 |  | 1.000 |  | 1.000 |
| Independence model | .000 | .000 | .000 | .000 | .000 |

##### Parsimony-Adjusted Measures

| Model | PRATIO | PNFI | PCFI |
| --- | --- | --- | --- |
| Default model | .795 | .773 | .776 |
| Saturated model | .000 | .000 | .000 |
| Independence model | 1.000 | .000 | .000 |

##### NCP

| Model | NCP | LO 90 | HI 90 |
| --- | --- | --- | --- |
| Default model | 400.041 | 335.441 | 472.123 |
| Saturated model | .000 | .000 | .000 |
| Independence model | 16634.517 | 16212.879 | 17062.439 |

##### FMIN

| Model | FMIN | F0 | LO 90 | HI 90 |
| --- | --- | --- | --- | --- |
| Default model | .448 | .388 | .325 | .457 |
| Saturated model | .000 | .000 | .000 | .000 |
| Independence model | 16.194 | 16.119 | 15.710 | 16.533 |

##### RMSEA

| Model | RMSEA | LO 90 | HI 90 | PCLOSE |
| --- | --- | --- | --- | --- |
| Default model | .079 | .072 | .086 | .000 |
| Independence model | .455 | .449 | .460 | .000 |

##### AIC

| Model | AIC | BCC | BIC | CAIC |
| --- | --- | --- | --- | --- |
| Default model | 520.041 | 520.838 | 663.307 | 692.307 |
| Saturated model | 182.000 | 184.503 | 631.560 | 722.560 |
| Independence model | 16738.517 | 16738.874 | 16802.740 | 16815.740 |

##### ECVI

| Model | ECVI | LO 90 | HI 90 | MECVI |
| --- | --- | --- | --- | --- |
| Default model | .504 | .441 | .574 | .505 |
| Saturated model | .176 | .176 | .176 | .179 |
| Independence model | 16.219 | 15.811 | 16.634 | 16.220 |

##### HOELTER

| Model | HOELTER .05 | HOELTER .01 |
| --- | --- | --- |
| Default model | 182 | 203 |
| Independence model | 7 | 7 |

##### Execution time summary

| Minimization: | .015 |
| --- | --- |
| Miscellaneous: | .313 |
| Bootstrap: | .000 |
| Total: | .328 |
